# The limiting factors and regulatory processes that control the environmental responses of C_3_, C_3_–C_4_ intermediate, and C_4_ photosynthesis

**DOI:** 10.1007/s00442-021-05062-y

**Published:** 2021-10-29

**Authors:** Jennifer E. Johnson, Christopher B. Field, Joseph A. Berry

**Affiliations:** 1 Department of Global Ecology, Carnegie Institution for Science, 260 Panama Street, Stanford, CA 94305 USA; 2 Stanford Woods Institute for the Environment, Stanford University, 473 Via Ortega, Stanford, CA 94305 USA

**Keywords:** Cytochrome b_6_f, Rubisco, Glycine decarboxylase, CO_2_ compensation point, Temperature

## Abstract

Here, we describe a model of C_3_, C_3_–C_4_ intermediate, and C_4_ photosynthesis that is designed to facilitate quantitative analysis of physiological measurements. The model relates the factors limiting electron transport and carbon metabolism, the regulatory processes that coordinate these metabolic domains, and the responses to light, carbon dioxide, and temperature. It has three unique features. First, mechanistic expressions describe how the cytochrome b_6_f complex controls electron transport in mesophyll and bundle sheath chloroplasts. Second, the coupling between the mesophyll and bundle sheath expressions represents how feedback regulation of Cyt b_6_f coordinates electron transport and carbon metabolism. Third, the temperature sensitivity of Cyt b_6_f is differentiated from that of the coupling between NADPH, Fd, and ATP production. Using this model, we present simulations demonstrating that the light dependence of the carbon dioxide compensation point in C_3_–C_4_ leaves can be explained by co-occurrence of light saturation in the mesophyll and light limitation in the bundle sheath. We also present inversions demonstrating that population-level variation in the carbon dioxide compensation point in a Type I C_3_–C_4_ plant, *Flaveria* *chloraefolia*, can be explained by variable allocation of photosynthetic capacity to the bundle sheath. These results suggest that Type I C_3_–C_4_ intermediate plants adjust pigment and protein distributions to optimize the glycine shuttle under different light and temperature regimes, and that the malate and aspartate shuttles may have originally functioned to smooth out the energy supply and demand associated with the glycine shuttle. This model has a wide range of potential applications to physiological, ecological, and evolutionary questions.

**Supplementary Information:**

The online version contains supplementary material available at 10.1007/s00442-021-05062-y.

## Introduction

At present, the major patterns in the ecology and evolution of C_3_ and C_4_ plants are attributed to the physiological differences between the C_3_ and C_4_ photosynthetic pathways. The foundation for this theory is the observation that the two pathways translate into distinct physiological advantages and disadvantages under different environmental conditions (Berry 1975; Ehleringer and Björkman 1977). C_3_ photosynthesis provides advantages over C_4_ photosynthesis under low light intensities, high carbon dioxide levels, and cool temperatures, while C_4_ photosynthesis provides advantages over C_3_ photosynthesis under high light intensities, low carbon dioxide levels, and warm temperatures (Berry and Björkman 1980; Berry and Downton 1982; Pearcy and Ehleringer 1984). These physiological trade-offs are thought to be the basis of the evolutionary rise of C_4_ plants in ancient environments characterized by lower atmospheric carbon dioxide (Monson 1989a; Ehleringer and Monson 1993; Ehleringer et al. 1997; Osborne and Beerling 2006; Tipple and Pagani 2007; Osborne and Sack 2012). They are also thought to be the basis of the ecological dominance of C_4_ plants in modern environments characterized by high light intensities, warm temperatures, and a moderate amount of warm season precipitation (Ehleringer 1978; Ehleringer and Monson 1993; Collatz et al. 1998; Still et al. 2003). However, it is not yet clear how to reconcile these ideas with the physiology, ecology, and evolution of plants that use C_3_–C_4_ intermediate photosynthesis.

On the one hand, a number of lines of evidence suggest that C_3_–C_4_ intermediate photosynthesis represents an intermediate stage in evolutionary transitions between C_3_ and C_4_ photosynthesis (Kennedy and Laetsch 1974; Christin et al. 2011; Sage et al. 2014; Stata et al. 2019; Lyu et al. 2021). On the other hand, no ecological conditions have yet been identified under which C_3_–C_4_ intermediate photosynthesis provides C_3_–C_4_ plants with unique physiological advantages over C_3_ as well as C_4_ plants (Monson et al. 1984, 1986; Peisker 1986; von Caemmerer 1989; Monson 1989a, 2003; Ehleringer and Monson 1993; Ehleringer et al. 1997; Sage et al. 2012, 2018; Heckmann et al. 2013; Christin and Osborne 2014; Lundgren and Christin 2017). The simplest way to reconcile these observations would be to posit that there are some ecological conditions under which C_3_–C_4_ intermediate photosynthesis can represent an evolutionarily stable strategy (Maynard Smith and Price 1973), and the exact nature of these conditions simply remains to be elucidated. But this may or may not be the right interpretation. At the heart of this puzzle is the question: what are the relative advantages and disadvantages of C_3_, C_3_–C_4_ intermediate, and C_4_ photosynthesis?

We submit that to answer this question, it is necessary to directly confront the current conceptual models of the C_3_, C_3_–C_4_ intermediate, and C_4_ pathways with experimental measurements. Towards this end, the objective of this paper is to describe a general model of C_3_, C_3_–C_4_ intermediate, and C_4_ photosynthesis that is designed to facilitate quantitative analysis of physiological measurements. This model uses the approach introduced by Johnson and Berry (2021) to describe how the overall photosynthetic responses to light, carbon dioxide, and temperature emerge from the factors limiting electron transport and carbon metabolism and the regulatory processes that coordinate these metabolic domains. In the remainder of the Introduction, we review current understanding of C_3_, C_4_, and C_3_–C_4_ intermediate photosynthesis and outline the philosophy that has guided the development of this modeling framework. In Model Development we then introduce the key features of this framework and in Model Applications we present example applications to the interpretation of physiological measurements.

### The C_3_ photosynthetic pathway

The current understanding of C_3_ photosynthesis began to be assembled more than a century ago. In the early 1900s, it was established that C_3_ photosynthesis involves the coordinated operation of two metabolic domains with different sensitivities to light, carbon dioxide, and temperature (Blackman 1905). During the 1910–1920s, it was inferred that at low light intensities the overall process is controlled by ‘a photochemical reaction’ with low temperature sensitivity, whereas at high light intensities the control shifts to a ‘purely chemical reaction’ with much higher temperature sensitivity (Emerson 1929). During the 1930–1960s, the nature of these processes was pursued. It was shown that the metabolic domain in control at low light is the electron transport system, and that it operates in two major modes. One is a linear electron flow that involves two photochemical reactions, splits water, and leads to the net production of oxygen, Fd, NADPH, and ATP (Duysens et al. 1961). The other is a cyclic electron flow that only involves one photochemical reaction and leads to the net production of ATP (Tagawa et al. 1963). The pigment-protein complexes mediating the photochemical reactions are now known as Photosystem II (PS II; EC 1.10.3.9) and Photosystem I (PS I; EC 1.97.1.12). In parallel, it was shown that the metabolic domain in control at high light is carbon metabolism, and that it also operates in two major modes: a photosynthetic carbon reduction cycle which fixes CO_2_ (Calvin and Benson 1948) and a photosynthetic carbon oxidation cycle which fixes O_2_ (Tregunna et al. 1966). During the late 1960s and early 1970s, the sites of the primary rate-limitations were identified within both metabolic domains. The primary rate-limiting factor within the electron transport system was found to be the Cytochrome b_6_f complex (Cyt b_6_f; EC 7.1.1.6), which participates in linear as well as cyclic electron flow (Stiehl and Witt 1969). The primary rate-limiting factor within carbon metabolism was found to be Ribulose-1,5-bisphosphate carboxylase/oxygenase (Rubisco; EC 4.1.1.39), which participates in the photosynthetic carbon reduction and oxidation cycles (Ogren and Bowes 1971; Bowes et al. 1971; Laing et al. 1974). During the 1980s and 1990s, advances were made in identifying the regulatory processes that mediate flux in between these limits. It was established that under saturating light there is downregulation of many of the membrane-bound enzymes in the electron transport system, including Cyt b_6_f, whereas under limiting light there is downregulation of many of the stromal enzymes involved in carbon metabolism, including Rubisco (Heber et al. 1986; Weis et al. 1987; Harbinson et al. 1990). In combination, these insights underpin current conceptual understanding of how the C_3_ photosynthetic pathway operates as an integrated system in dynamic environments.

The development of quantitative models of C_3_ photosynthesis proceeded in parallel with the development of conceptual understanding. Initially, the light response of photosynthesis was described as having a domain where the photosynthetic rate increases linearly with light, and a sharp transition to another domain where the CO_2_ supply becomes limiting (Blackman 1905). A rectangular hyperbolic function was then introduced to provide a smoother transition from the light-limited and CO_2_-limited parts of the response (Rabinowitch 1951). This was eventually supplanted by a more flexible non-rectangular hyperbola, which included an additional parameter to describe the degree of curvature of the light response (Thornley 1976). The non-rectangular hyperbolic description of the light response was then linked to a mechanistic expression for Rubisco based on competitive inhibition between CO_2_ and O_2_ (Farquhar et al. 1980a). This formulation is effective at approximating the environmental responses of gas-exchange, but incorrectly predicts that the activity of the electron transport system limits the activity of carbon metabolism under saturating light and saturating CO_2_. To address this issue, another model was developed that links a linear, Blackman-type light response to the mechanistic expression for Rubisco (Collatz et al. 1991). This framework correctly predicts that the activity of carbon metabolism limits the activity of the electron transport system under saturating light and saturating CO_2_. Since it is effective at describing the states of regulation under different environmental conditions, it was also extended to simulate chlorophyll fluorescence (van der Tol et al. 2014). While this model performs well when it can be calibrated directly, it is still empirical and the predictive skill degrades outside the calibration domain. To address this issue, we have recently developed a new model that links a Cyt b_6_f-based description of the light response to the Rubisco-based description of the CO_2_ response (Johnson and Berry 2021). By describing the light response mechanistically rather than empirically, the model can be used to infer the state of regulation under different light intensities and CO_2_ levels. This permits more accurate and efficient simulation of gas-exchange, chlorophyll fluorescence, and other optical probes of electron transport. It also provides a new basis for analyzing how the temperature sensitivities of the electron transport system contribute to the overall temperature response of photosynthesis. The latter is a key priority both for understanding the C_3_ pathway itself, and for understanding the relative advantages and disadvantages of the C_3_ relative to the C_4_ pathway.

### The C_4_ photosynthetic pathway

Insights into the differences between C_3_ and C_4_ photosynthesis began to emerge during the 1960s. During this period, it was established that the C_3_ pathway takes place entirely in leaf mesophyll cells, but the C_4_ pathway involves both the mesophyll and bundle sheath. It was found that in C_4_ photosynthesis, CO_2_ from the mesophyll is initially fixed into oxaloacetate by PEP carboxylase (PEPC; EC 4.1.1.31) and oxaloacetate is converted to malate and/or aspartate (Kortschak et al. 1965; Slack and Hatch 1967). The organic acids were then found to be shuttled into the bundle sheath, where Rubisco is situated (Björkman and Gauhl 1969). During the 1970s, it was established that the NADP-malic enzyme (NADP-ME; EC 1.1.1.40), NAD-malic enzyme (NAD-ME; EC 1.1.1.39), and/or PEP carboxykinase (PEPCK; EC 4.1.1.49) decarboxylates the organic acids in the bundle sheath, releasing CO_2_ in the vicinity of Rubisco (Hatch et al. 1975). In combination, these observations indicated that the primary rate-limiting factor in mesophyll carbon metabolism is PEP carboxylase, while that in bundle sheath carbon metabolism is Rubisco. During the 1980s, the relationship between cell type-specific patterns of carbon metabolism and electron transport was pursued. It was found that the distinct energetic requirements of mesophyll and bundle sheath carbon metabolism are supported by modifications in linear and cyclic flow within each cell type, and that these vary depending on the decarboxylation pathway (Chapman et al. 1980; Hatch 1987). This suggested that the efficient operation of the C_4_ pathway requires cell type-specific balancing of the absorption cross-sections of PS I and PS II in relation to the primary kinetic bottleneck at Cyt b_6_f. During the 1990s, there were initial advances in understanding the regulatory processes that coordinate flux between the limits set by Cyt b_6_f, PEPC, and Rubisco. In addition to the forms of regulation evident in the C_3_ cycle, it was established that under limiting light there is downregulation of PEPC that is coordinated with that of other enzymes in the C_4_ cycle (Furbank and Taylor 1995; Chollet et al. 1996; Leegood and Walker 1999). This reinforced the earlier idea that regulation of rate-limiting steps is the general way of maintaining coordination between electron transport and carbon metabolism. However, the conceptual understanding of how the C_4_ photosynthetic pathway operates as an integrated system in dynamic environments has remained relatively less clear and less comprehensive than analogous understanding of the C_3_ photosynthetic pathway. Due to the complexity of the C_4_ system, there is a need and an opportunity for modeling to contribute here.

The development of quantitative models of C_4_ photosynthesis has been closely linked to the development of quantitative models of C_3_ photosynthesis. In the late 1970s, a conceptual model was formulated that represented PEPC activity in the mesophyll as the rate-limiting step in the initial fixation of CO_2_, transport of organic acids and decarboxylation in the bundle sheath (Berry and Farquhar 1978). This model demonstrated that when the C_4_-cycle pumps CO_2_ into the bundle sheath faster than CO_2_ leaks out of the bundle sheath, the resulting increase in the bundle sheath CO_2_ relative to O_2_ competitively inhibits O_2_ fixation, allowing Rubisco to operate close to its maximal rate of CO_2_ fixation. Similar to the C_3_ models formulated around this time, this formulation is effective at approximating the environmental responses of gas-exchange, but incorrectly predicts that the activity of the electron transport system limits the activity of carbon metabolism under saturating light and saturating CO_2_. During the 1990s, another model was developed that addressed this issue by linking a linear, Blackman-type light response to mechanistic expressions for PEPC and Rubisco (Collatz et al. 1992). However, this model also intentionally simplified the kinetic descriptions of PEPC and Rubisco to facilitate applications to larger-scale environmental simulations (Sellers et al. 1996a,b). It was later extended to simulate chlorophyll fluorescence (van der Tol et al. 2014). In a parallel strand of C_4_ model development, the earlier non-rectangular hyperbolic expression for the light response was brought back in and was again linked to the complete kinetic expressions for PEPC and Rubisco (von Caemmerer and Furbank 1999; von Caemmerer 2000, 2021). In both of these strands of C_4_ model development, the potential linear electron transport of the leaf has been modeled as a whole and then partially allocated to the C_3_ and C_4_ cycles. This approach was designed to provide a simple way to accommodate the variable contributions of the different C_4_ decarboxylases and 3-phosphoglyceric acid export to the energy budget. A number of refinements of the stoichiometries associated with these processes have been proposed over the years (e.g., Furbank et al. 1990; Yin and Struik 2012, 2018, 2021; Bellasio 2017). However, the fundamental issue remains that all of these C_4_ models are missing a fully mechanistic description of the light response, and this limits their ability to clearly explain the coordination between electron transport and carbon metabolism. This is a key priority for model development, and one that we will tackle here by progressively building up the components of the full C_4_ pathway from their foundation in the C_3_–C_4_ intermediate pathways.

### The C_3_–C_4_ intermediate photosynthetic pathways

When C_3_–C_4_ intermediate photosynthesis was originally discovered in the 1970s, it was clear that C_3_–C_4_ intermediate plants exhibited a sensitivity to O_2_ that was intermediate between the sensitivities of C_3_ and C_4_ plants, but it was not clear what mechanism(s) were responsible for this (Kennedy and Laetsch 1974). Two conceptual models were considered: one in which the intermediate sensitivity to O_2_ was due to low levels of C_4_-cycle activity, and one in which it was due to refixation of CO_2_ released from the photosynthetic carbon oxidation cycle (Monson et al. 1984). Before a mechanism was identified for the refixation of CO_2_ released from the photosynthetic carbon oxidation cycle, a quantitative model of C_3_–C_4_ intermediate photosynthesis was developed based on the assumption that the physiological characteristics of C_3_–C_4_ intermediate plants were generated solely by low levels of C_4_-cycle activity (i.e., via malate and/or aspartate shuttling; Peisker 1984, 1986; Peisker and Bauwe 1984). Following the discovery that the P-protein of the glycine decarboxylase (GDC; EC 1.4.4.2) complex was localized to the bundle sheath in C_3_–C_4_ intermediate plants, it became clear that the physiological characteristics of some C_3_–C_4_ intermediate plants might be generated solely by recycling of CO_2_ released from the photosynthetic carbon oxidation cycle within the bundle sheath (Hylton et al. 1988; i.e., via glycine shuttling; Rawsthorne et al. 1988a, b). Based on this conceptual model, von Caemmerer (1989, 1992) developed a new quantitative model to represent the glycine shuttle. Later, von Caemmerer (2000) synthesized the expressions developed by Peisker and Bauwe (1984), Peisker (1986), von Caemmerer (1989), and von Caemmerer (1992) into equations that could represent Type I, Type II, or C_4_-like C_3_–C_4_ intermediate photosynthesis (Table 1). In the equation set described by von Caemmerer (2000), the basis of the glycine shuttle is the localization of some of the Rubisco activity and some or all of the GDC activity in the bundle sheath rather than in the mesophyll. Under environmental conditions that exacerbate Rubisco oxygenase activity in the mesophyll, this configuration leads to: (i) net production of glycine in the mesophyll; (ii) net diffusion of glycine into the bundle sheath; (iii) decarboxylation of that glycine within the bundle sheath, (iv) an increase in the bundle sheath CO_2_/O_2_ ratio, and (v) a corresponding increase in the efficiency of CO_2_ fixation by the bundle sheath Rubisco. Under environmental conditions that exacerbate Rubisco oxygenase activity in the bundle sheath, (i)–(v) are reversed. As a result of this environmental sensitivity, there is not a fixed energetic cost or benefit associated with the glycine shuttle. Instead, the glycine shuttle tends to confer a net energetic benefit over the C_3_ pathway under conditions that promote Rubisco oxygenase activity in the mesophyll, and a net energetic cost under conditions that suppress such activity.

**Table 1:** Functional distinctions between C_3_, C_3_–C_4_, and C_4_ photosynthesis

Pathway	Enzyme localization					Bundle sheath CO_2_ source		
	PS I & II	Cyt b_6_f	Rubisco	GDC	PEPC	Glycine shuttle	Malate shuttle	Aspartate shuttle
C_3_	M	M	M	M	–	–	–	–
Proto-Kranz C_3_–C_4_	M, BS	M, BS	M, BS	M, BS	–	Low activity	–	–
Type I C_3_–C_4_	M, BS	M, BS	M, BS	BS	–	High activity	–	–
Type II C_3_–C_4_	M, BS	M, BS	M, BS	BS	M	High activity	Low activity	Low activity
C_4_-like C_3_–C_4_	M, BS	M, BS	M, BS	BS	M	Low activity	Variable activity	Variable activity
NADP-ME C_4_	M, BS	M, BS	BS	BS	M	–	High activity	Variable activity
NAD-ME C_4_	M, BS	M, BS	BS	BS	M	–	Variable activity	High activity
PEPCK C_4_	M, BS	M, BS	BS	BS	M	–	Variable activity	Variable activity

Traditionally, the categorization of C_3_–C_4_ and C_4_ plants into discrete ‘types’ has been used to emphasize general patterns of functional similarities and differences. However, it is important to recognize that many biochemical and anatomical attributes of these plants exhibit continuous variation. *PS* *I* Photosystem I, *PS* *II* Photosystem II, *Cyt* *b*_*6*_*f* Cytochrome b_6_f complex, *Rubisco* Ribulose-15-bisphosphate carboxylase-oxygenase, *GDC* Glycine decarboxylase complex, *PEPC* PEP carboxylase, *NADP-ME* NADP-malic enzyme, *NAD-ME* NAD-malic enzyme, *PEPCK* PEP carboxykinase, *M* mesophyll, *BS* bundle sheath

At present, it is unclear whether this model is correct. Forward simulations based on these equations have generated predictions that Type I C_3_–C_4_ plants should exhibit higher rates of net CO_2_ assimilation than C_3_ plants under typical midday conditions (i.e., saturating light, moderate leaf temperatures, and modern atmospheric CO_2_ and O_2_ levels; von Caemmerer 1989, 2000; Schuster and Monson 1990; Monson and Rawsthorne 2000; Heckmann et al. 2013; Mallmann et al. 2014; Way et al. 2014; Bellasio and Farquhar 2019). All else being equal, there is a clear theoretical basis for expecting that such relative advantages in the net CO_2_ assimilation rates should translate into corresponding advantages in the photosynthetic resource-use efficiencies (Field 1983; Field et al. 1983; Field and Mooney 1986; Monson 1989b; Schuster and Monson 1990; Monson and Rawsthorne 2000; Christin and Osborne 2014; Way et al. 2014; Lundgren 2020; Sundermann et al. 2021). However, Type I C_3_–C_4_ plants have not consistently exhibited higher photosynthetic rates, light-use efficiencies, water-use efficiencies, or nitrogen-use efficiencies than C_3_ plants when measurements have been made under these conditions (Brown and Brown 1975; Brown and Simmons 1979; Bolton and Brown 1980; Ehleringer and Pearcy 1983; Henning and Brown 1986; Monson et al. 1986; Fladung and Hesselbach 1989; Monson 1989b; Krall et al. 1991; Ku et al. 1991; Monson and Rawsthorne 2000; Huxman and Monson 2003; Voznesenskaya et al. 2007; Vogan et al. 2007; Pinto et al. 2011, 2014; Vogan and Sage 2011, 2012; Khoshravesh et al. 2016; Lundgren et al. 2016). From a quantitative perspective, there are two explanations for the discrepancy between the observations and predictions: either (i) there is a fundamental problem with the structure of the model of C_3_–C_4_ photosynthesis, or (ii) the structure of the model is correct, but there is a fundamental problem with the parameterizations used for the simulations. For example, it has been suggested that the strong light dependence of the CO_2_ compensation point may indicate that C_3_–C_4_ photosynthesis does not work exactly as described above (von Caemmerer 2000). The most straightforward way to differentiate between this type of structural error versus a parameterization error is to fit the C_3_–C_4_ model to physiological measurements, objectively evaluate the quality of the fit between the model and the measurements, and compare the measurement-derived parameter set to the parameter set specified for the simulations. With this approach, the quality-of-fit statistics can be used to identify problems with model structure (i.e., testing the first hypothesis), and the inverse parameter estimates can be used to identify problems with model parameterization (i.e., testing the second hypothesis). To enable this mode of analysis, we will rely on the approach introduced by Johnson and Berry (2021) and described below.

### A general framework for modeling C_3_, C_3_–C_4_, and C_4_ photosynthesis

**Fig. 1:**
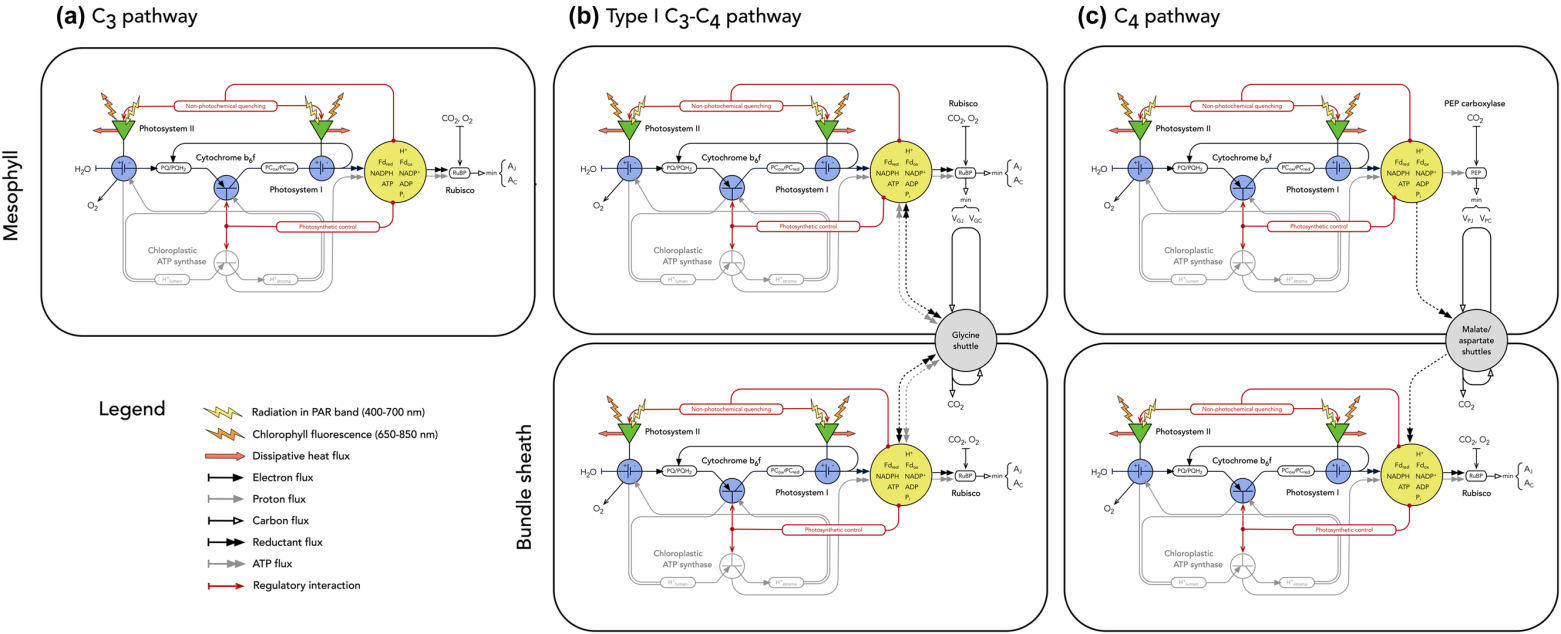
Circuit diagrams for C_3_, C_3_–C_4_ intermediate, and C_4_ photosynthesis. **a** The C_3_ case follows Johnson and Berry (2021). **b** The Type I C_3_–C_4_ intermediate case represents both the mesophyll and bundle sheath, and their coupling through the glycine shuttle. **c** The C_4_ case represents the localization of all Rubisco activity to the bundle sheath, and the delivery of CO_2_ from the mesophyll to bundle sheath through the malate and/or aspartate shuttles. Details are provided in text

Although inverse fitting has started to become established as an approach for interpreting physiological measurements of C_3_ and C_4_ plants, it is still not widely used and has not yet been applied to C_3_–C_4_ plants (e.g., Yin et al. 2009, 2011; Bellasio et al. 2016; Zhou et al. 2019). The main reason for this is that none of the current C_3_, C_3_–C_4_, and C_4_ models are formulated in a way that is ideal for fitting to experimental data. On the one hand, in the type of models that are simple enough to be fit directly to data and that we have discussed here, a large fraction of the key parameters are empirical (e.g., the *θ* and *J*_max_ parameters describing the curvature and asymptote of the light response, respectively). Since there is no way to independently evaluate the fitted values of the empirical parameters in these frameworks, it is difficult to differentiate between problems with model structure versus model parameterization. On the other hand, in more complex models where a larger fraction of the parameters are mechanistic, there are simply too many free variables to constrain with typical experimental observations (e.g., Laisk and Edwards 2000; Laisk et al. 2009; Zhu et al. 2013; Wang et al. 2014, 2017, 2021; Morales et al. 2018). Since there is no way to rigorously confront the hypotheses in these frameworks with data, it is also difficult to differentiate between problems with model structure versus model parameterization. To address these challenges and opportunities, we have developed a general model of C_3_, C_3_–C_4_, and C_4_ photosynthesis that is designed to facilitate inverse analysis of physiological measurements (Fig. 1).

The new model is organized around three design criteria. *First,* *this* *framework* *is* *designed* *to* *describe* *the* *overall* *photosynthetic* *process* *at* *the* *leaf-level* *when* *it* *has* *achieved* *a* *fully* *reversible* *steady-state* *with* *the* *measuring* *environment.* At this spatial and temporal scale, the dynamics of photosynthesis are much simpler than they are within particular sub-systems and/or during transient adjustments because they become keyed to the boundary conditions imposed by energy, mass, and charge balance. This makes this scale a tractable starting point for mathematical analysis. *Second,* *this* *framework* *is* *designed* *to* *link* *the* *photosynthetic* *process* *to* *the* *full* *suite* *of* *observable* *quantities* *that* *can* *be* *evaluated* *via* *leaf-level* *measurements.* Most current frameworks for modeling leaf-level photosynthesis are organized around trace gas concentration and/or isotope ratio measurements. Here, we have formulated a model that can also communicate with a diverse array of fluorescence- and absorbance-based measurements. *Third,* *this* *framework* *is* *designed* *to* *explain* *the* *environmental,* *biochemical,* *and* *anatomical* *factors* *that* *control* *the* *patterns* *of* *photosynthetic* *performance.* In formulating this model, we have avoided empirical descriptions that rely on statistical coefficients without precise physical meaning, as well as putatively mechanistic descriptions that are untestable. Instead, we have articulated hypotheses about the specific mechanisms that give rise to the observed patterns, and we have done so in a way that permits these hypotheses to be evaluated with independent measurements.

The new model brings together the description of C_3_ electron transport from Johnson and Berry (2021) with the descriptions of C_3_, C_3_–C_4_, and C_4_ carbon metabolism from Berry and Farquhar (1978), Farquhar et al. (1980a), Farquhar and von Caemmerer (1981), von Caemmerer and Farquhar (1981), Peisker and Bauwe (1984), Peisker (1986), von Caemmerer (1989), Collatz et al. (1991), Collatz et al. (1992), von Caemmerer and Furbank (1999), von Caemmerer (2000), von Caemmerer et al. (2009), von Caemmerer (2013), van der Tol et al. (2014), von Caemmerer (2020, 2021). The resulting framework describes the steady-state responses of C_3_, C_3_–C_4_, and C_4_ photosynthesis to variation in light, carbon dioxide, and temperature. While the model can generate simulations over arbitrarily wide ranges of measurement light intensities, carbon dioxide levels, and leaf temperatures, it does not represent stress responses. As a result, the descriptions of regulatory interactions are only accurate within the ranges of conditions that leaves have acclimated to during growth. The equations that correspond to the schematic in Fig. 1 are presented in Appendices I-V in the Electronic Supplementary Material. To draw attention to the core aspects of model structure, we focus on the C_3_, Type I C_3_–C_4_, and NADP-ME C_4_ cases (Table 1), and we illustrate these cases using a simplified model parameterization (Table 2). In the next section (Model Development), we discuss the series of three steps in model development, emphasizing the rationale for and theory underlying each step. In the subsequent section (Model Application), we then demonstrate how these developments come together in a way that enables the model to be applied for the quantitative interpretation of physiological measurements.

**Table 2:** Input parameters for C_3_, Type I C_3_–C_4_, and C_4_ simulations

Category	Symbol	Values	Units	Description
Environmental	*Q*	0–2400	umol PPFD m^−2^ s^−1^	Photosynthetically active radiation
variables	*T*	10–40	°C	Leaf temperature
	*C*_*m*_	0–1000	$\mu$bar CO_2_	Partial pressure of CO_2_ in mesophyll chloroplasts
	*O*_*m*_	209	mbar O_2_	Partial pressure of O_2_ in mesophyll chloroplasts
	*P*	1	bar	Total pressure
Electron transport	*α*	0.85	mol mol^−1^	Total leaf absorbance to PAR
variables	*α*_1_/*α*	48, 48, 53	%	PS I fraction of total leaf absorbance
	*α*_2_/*α*	52, 52, 47	%	PS II fraction of total leaf absorbance
	*α*_*m*_/*α*	100, 95, 60	%	Mesophyll fraction of total leaf absorbance
	*α*_*s*_/*α*	0, 5, 40	%	Bundle sheath fraction of total leaf absorbance
	*V*_*max*_ (*CB6F*)	175	$\mu$mol e- m^−2^ s^−1^	Maximum activity of Cyt b_6_f
	*V*_*mmax*_/*V*_*max*_ (*CB6F*)	100, 95, 60	%	Mesophyll fraction of Cyt b_6_f
	*V*_*smax*_/*V*_*max*_ (*CB6F*)	0, 5, 40	%	Bundle sheath fraction of Cyt b_6_f
Carbon metabolism	*V*_*max*_ (*RUB*)	50, 50, 30	$\mu$mol CO_2_ m^−2^ s^−1^	Maximum carboxylase activity of Rubisco
variables	*V*_*mmax*_/*V*_*max*_ (*RUB*)	100, 90, 0	%	Mesophyll fraction of Rubisco
	*V*_*smax*_/*V*_*max*_ (*RUB*)	0, 10, 100	%	Bundle sheath fraction of Rubisco
	*V*_*max*_ (*PEPC*)	0, 0, 60	$\mu$mol CO_2_ m^−2^ s^−1^	Maximum activity of PEPC
	*V*_*mmax*_/*V*_*max*_ (*PEPC*)	0, 0, 100	%	Mesophyll fraction of PEPC
	*V*_*smax*_/*V*_*max*_ (*PEPC*)	0, 0, 0	%	Bundle sheath fraction of PEPC
	*g*_*bs*_	0.003	mol CO_2_ m^−2^ s^−1^	Bundle sheath conductance to CO_2_
	*R*_*d*_	0.010	%	Dark respiration scaled to V_max_ Rubisco
Electron transport	*K*_*F1*_*,* *K*_*F2*_	0.05	ns^−1^	Rate constant for fluorescence at PS I & PS II
constants	*K*_*D1*_*,* *K*_*D2*_	0.55	ns^−1^	Rate constant for const. heat loss at PS I & PS II
	*K*_*P1*_	14.5	ns^−1^	Rate constant for photochemistry at PS I
	*K*_*P2*_	4.5	ns^−1^	Rate constant for photochemistry at PS II
	*K*_*U2*_	2.0	ns^−1^	Rate constant for excitation sharing at PS II
	*k*_*q*_	300	mol PQH_2_ mol^−1^ sites s^−1^	Catalytic constant for PQH_2_ for Cyt b_6_f
	*n*_*L*_	0.75	mol ATP mol^−1^ e-	Coupling efficiency of linear electron flow
	*n*_*C*_	1.00	mol ATP mol^−1^ e-	Coupling efficiency of cyclic electron flow
Carbon metabolism	*k*_*c*_	3.6	mol CO_2_ mol^−1^ sites s^−1^	Catalytic constant for CO_2_ for Rubisco
constants	*k*_*o*_	0.9	mol O_2_ mol^−1^ sites s^−1^	Catalytic constant for O_2_ for Rubisco
	*K*_*c*_	260	$\mu$bar	Michaelis constant for CO_2_ for Rubisco
	*K*_*o*_	179	mbar	Michaelis constant for O_2_ for Rubisco
	*K*_*p*_	80	$\mu$bar	Michaelis constant for CO_2_ for PEPc
	*D*_*c*_	1*.*9 *·* 10^*−*9^	m^2^ s^−1^	Diffusivity of CO_2_ in air
	*H*_*c*_	59*.*4 *·* 10^*−*5^	bar^−1^	Solubility of CO_2_ in water
	*D*_*o*_	2*.*4 *·* 10^*−*9^	m^2^ s^−1^	Diffusivity of O_2_ in air
	*H*_*o*_	2*.*2 *·* 10^*−*5^	bar^−1^	Solubility of O_2_ in water

We have used an intentionally simplified parameterization to facilitate interpretation of the simulations. For most parameters, only a single value is given and this is applied to all three pathways. For the pathway-specific parameterizations, values are given for C_3_, Type I C_3_–C_4_, and C_4_ cases, respectively. The parameterization of constants related to carbon metabolism follows von Caemmerer (2000), and the parameterization of constants related to electron transport follows Johnson and Berry (2021). The values given for the physiological variables and all of the constants correspond to a reference temperature of 25 °C. The scaling of temperature-sensitive parameters follows the approach described in the Model Development section, where the activation term from *J*_max_ is assigned to *V*_max_ of Cyt b_6_f and the deactivation term from *J*_max_ is assigned to the *n*_L_ and *n*_*C*_ parameters that describe the efficiency of coupling between NADPH, Fd, and ATP production

## Model development

In this model, we use the mechanistic description of electron transport from Johnson and Berry (2021) to represent the potential electron transport capacities of the mesophyll chloroplasts and bundle sheath chloroplasts separately. This creates a foundation for analyzing the mechanisms of energy sharing between the mesophyll and bundle sheath. We begin by introducing the Cyt b_6_f-based expression for electron transport and replicating it for the mesophyll and bundle sheath. Next, we discuss a hierarchical solution to the model that permits the mesophyll and bundle sheath to transition independently between limitation by electron transport and carbon metabolism. Finally, we turn to the formulation of the temperature response functions for electron transport and carbon metabolism.

### Limits and regulation of electron transport

What controls the light response of steady-state photosynthesis? To answer this question in a general way, we have extended a mechanistic framework for modeling steady-state electron transport in the C_3_ case (Fig. 1a) to the C_3_–C_4_ and C_4_ cases (Figs. 1b and c). This framework is organized around linear electron flow (LEF). In this process, light that is absorbed by PS II and PS I is used to split H_2_O and drive electrons through the intersystem chain to form reductant (Fd and NADPH). Within the intersystem chain, the light-driven electron flow is coupled to proton pumping at Cyt b_6_f, and the resulting proton motive force is used to drive ATP production at the ATP synthase. The Fd, NADPH, and ATP are then consumed by C_3_ and/or C_4_ carbon metabolism (i.e., Eqs. 1–10). Our approach to modeling LEF is based on the observation that Cyt b_6_f is the primary kinetic bottleneck in the electron transport system and is the target of a hierarchy of regulatory feedbacks stemming from carbon metabolism (e.g., see reviews by Kallas 2012; Schöttler and Tóth 2014; Finazzi et al. 2016; Tikhonov 2018; Simkin et al. 2019; Malone et al. 2021; Sarewicz et al. 2021). We developed experimental methods to estimate the in vivo maximum activity of Cyt b_6_f and identify the conditions under which feedback control of Cyt b_6_f is active or relaxed, and then used these approaches to relate the biochemical properties of Cyt b_6_f to the steady-state dynamics of photosynthesis in intact C_3_ leaves (Johnson and Berry 2021).

**Fig. 2:**
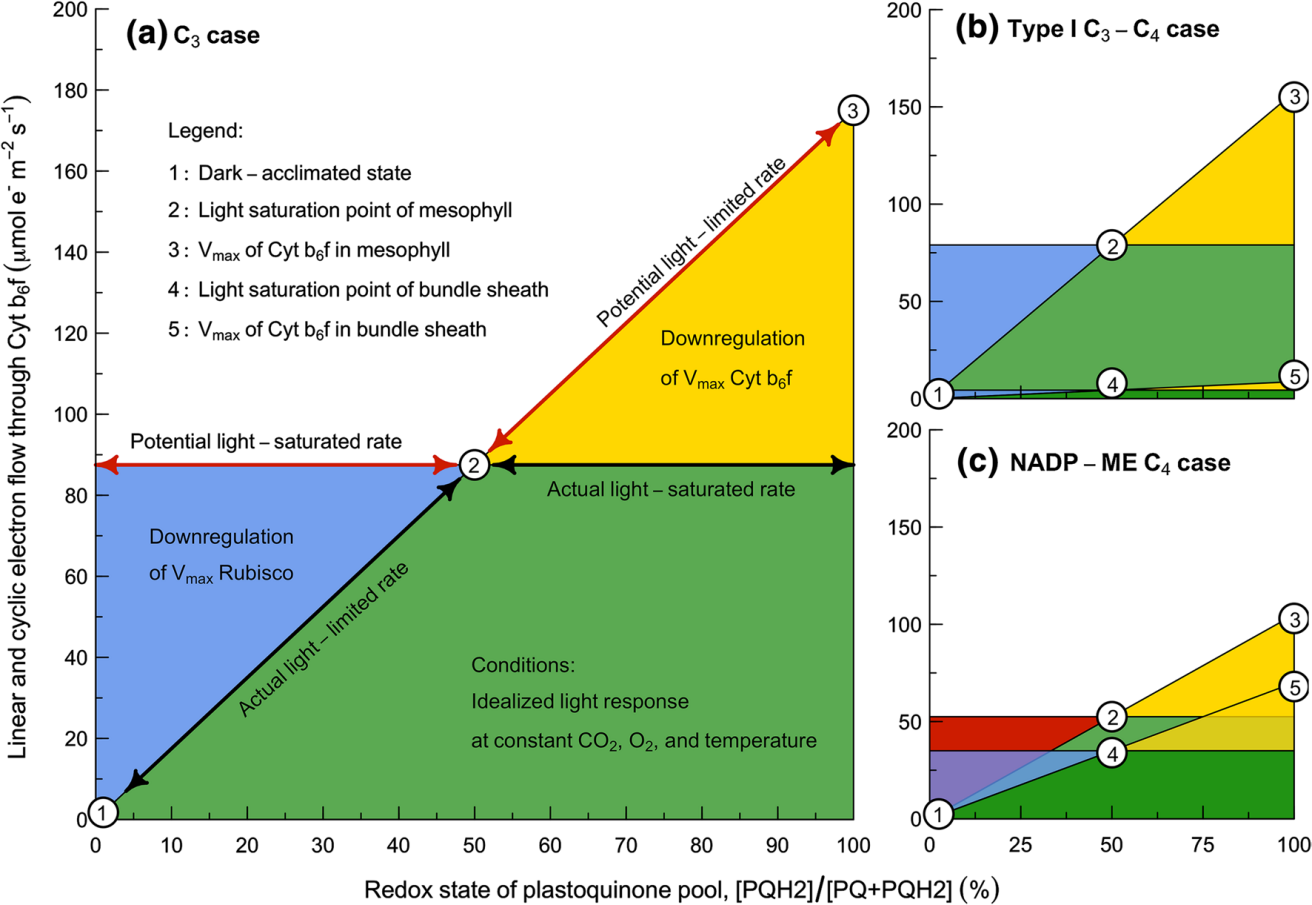
The maximum activity of Cyt b_6_f limits electron transport in the mesophyll and bundle sheath. For the **a** C_3_, **b** Type I C_3_–C_4_, and **c** C_4_ cases, the light-limited rates are defined by the sloping lines from the dark-acclimated state (i.e., point 1) to the *V*_max_ of Cyt b_6_f (i.e., point 3 in mesophyll and point 5 in bundle sheath), and the light-saturated rates are defined by the horizontal lines through the light-saturation points (i.e., point 2 in mesophyll and point 4 in bundle sheath). See text for discussions of the shaded regions. N.B., *x*- and *y*-axes are the same for all panels. *LEF* linear electron flow, *CEF1* cyclic electron flow around PS I, *PQ* plastoquinone, *PQH*_*2*_ plastoquinol, *V*_*max*_ maximum activity

Mathematically, this framework describes the potential rate of electron transport through Cyt b_6_f with a concise analytic expression that is a rectangular hyperbolic function of incident light intensity (i.e., Eqs. 11a, b), and a linear function of the redox state of plastoquinone (Fig. 2). This form of the electron transport response to light emerges from two regulated properties: (i) at the limit where light approaches zero, the initial slope is determined by the absorption cross-sections and maximum photochemical yields of PS I and PS II (Fig. 2a; dark-acclimated state); whereas (ii) at the limit where light goes to infinity, the asymptote is determined by the maximum activity of Cyt b_6_f (Fig. 2a; *V*_max_ of Cyt b_6_f in mesophyll). In between these limits, the sensitivity of electron transport to light declines progressively because the light-independent kinetic bottleneck at Cyt b_6_f causes an increasing fraction of the PS I and PS II reaction centers to accumulate in the closed state as the light-dependent excitation pressure on PS I and PS II increases. Due to the kinetic restriction at Cyt b_6_f, the closed state is reduced at PS II and oxidized at PS I, but in both cases represents a state where photochemistry cannot occur. This creates a fundamental trade-off between the rate and the efficiency of potential electron transport through Cyt b_6_f, and this trade-off structures the responses of photosynthesis to CO_2_, O_2_, and temperature.

As environmental conditions vary, regulatory interactions that center on Cyt b_6_f maintain coordination between electron transport and carbon metabolism (e.g., see reviews by Woodrow and Berry 1988; Foyer et al. 1990, 2012; Genty and Harbinson 1996; Baker et al. 2007; Cornic and Baker 2012; Tikkanen et al. 2012; Malone et al. 2021). For our purposes, it is sufficient to differentiate between two limiting states. We use ‘light-limited’ to refer to the metabolic state where electron transport is limiting carbon metabolism (i.e., Eqs. 11–16), and ‘light-saturated’ to refer to the metabolic state where carbon metabolism is limiting electron transport (i.e., Eqs. 17–20). Under any specific environmental condition, the actual state corresponds to the most limiting of the potential states (Fig. 2; upper bounds of green shaded regions). In the light-limited state, electron transport activity depends on the supply of substrate (reduced plastoquinone) and the *V*_max_ of the rate-limiting enzyme (Cyt b_6_f) (Fig. 2a–c; actual light-limited rate). Since the total light-driven electron flow is insufficient to satisfy the potential demand of the sinks for NADPH, Fd, and ATP, the activity of Rubisco and/or PEPC is downregulated to an extent that is determined by the availability of electron donors (Fig. 2a–c; blue and red shaded regions). In the light-saturated state, these patterns are reversed (Fig. 2a–c; actual light-saturated rate). Here, the activity of the sinks depends on the supply of substrate (CO_2_ and O_2_) and the *V*_max_ of the rate-limiting enzymes (Rubisco and/or PEPC). Since the total potential light-driven electron flow would exceed the capacity of the sinks, the activity of Cyt b_6_f is downregulated to an extent that is determined by the availability of electron acceptors (Fig. 2a–c; yellow shaded regions).

The definition of the light-limited and light-saturated states permits quantification of three forms of regulation: cyclic electron flow around PS I (CEF1), non-photochemical quenching of PS II (NPQ), and photosynthetic control of Cyt b_6_f (PC). In CEF1, light that is absorbed by PS I is used to drive electrons from the Fd and/or NADPH pools back into the intersystem chain and through Cyt b_6_f. Since the associated proton pumping can drive ATP formation, this pathway can be engaged to balance the energy supply from electron transport with the energy demand of carbon metabolism. In the steady-state, the partitioning between LEF and CEF1 is controlled by the excitation of the PS II versus PS I antennae. As sink demands for Fd, NADPH, and ATP vary, NPQ can be engaged to adjust the excitation distribution between PS II and PS I. Different forms of NPQ can be engaged under different conditions (e.g., state transitions (qT), chloroplast movements (qM), psbS-dependent (qE) and zeaxanthin-dependent (qZ) quenching). However, the family of NPQ processes does not dissipate all of the excess excitation from the antennae system under saturating light. To protect the system from photodamage, PC can be engaged. Photosynthetic control restricts linear electron flow through Cyt b_6_f to a rate that is balanced with the capacity of carbon metabolism to provide electron acceptors. Since all three of these regulatory interactions coordinate electron transport with carbon metabolism between the lumen and stroma of individual chloroplasts, they provide a foundation for analyzing how photosynthesis works when there is specialization of electron transport and carbon metabolism between populations of chloroplasts in the mesophyll and bundle sheath.

### Interactions between mesophyll and bundle sheath

While chloroplasts that are experiencing similar environmental conditions and have similar photosynthetic capacities are expected to transition synchronously between light-limited and light-saturated states, chloroplasts that are experiencing dissimilar environmental conditions and/or have different photosynthetic capacities are expected to transition asynchronously between light limitation and light saturation. To model photosynthesis in a way that captures these dynamics, the equation set needs to be solved differently than has typically been done to date. The C_3_–C_4_ model described by von Caemmerer (2000) and the C_4_ model described by von Caemmerer (2021) are based on expressions for pure states, i.e., conditions where the mesophyll and bundle sheath are both light-limited or both light-saturated. However, since the mesophyll and bundle sheath chloroplasts are segregated in distinct environments and since each population has distinct capacities for electron transport and for carbon metabolism, the mesophyll and bundle sheath have the potential to transition independently between light-limitation and light-saturation, creating mixed states (i.e., Eqs. 21–22). The possibility of mixed states occurring within the C_4_ pathway was originally hypothesized by Peisker and Henderson (1992), and it applies equally to the C_3_–C_4_ pathway. To simulate mixed states in a realistic way, the equation set needs to be solved in a way that respects two logical constraints: (i) the limiting state of the mesophyll controls the rate of glycine shuttling and malate shuttling to the bundle sheath, and therefore influences the bundle sheath environment, but (ii) the bundle sheath may or may not operate under the same limiting state as the mesophyll. To achieve this, we have developed a hierarchical approach to solving the equation set (i.e., Eqs. 23–25).

To evaluate the hierarchical solution, we tested the skill of the full model in simulating the light response of the CO_2_ compensation point ($\Gamma$). The CO_2_ compensation point is defined as the CO_2_ concentration at which there is no net CO_2_ assimilation. The $\Gamma$ values of C_3_ and C_4_ plants are nearly insensitive to light intensity, with the exception being at very low light intensities where there is a respiratory effect (Laing et al. 1974; Čatský and Tichá 1979; Peisker 1979; Farquhar et al. 1980a; Brooks and Farquhar 1985). In the C_3_ case, $\Gamma$ corresponds to a state where the CO_2_ uptake driven by Rubisco carboxylase activity is balanced by the CO_2_ loss driven by Rubisco oxygenase activity and mitochondrial (dark) respiration (i.e., from Eq. 5, *V*_cm_ = *V*_gm_ + *R*_m_). In the C_4_ case, $\Gamma$ corresponds to a state where the CO_2_ uptake driven by PEPC in the mesophyll is balanced by the CO_2_ loss driven by the diffusive leak out of the bundle sheath (i.e., from Eq. 5, *V*_pm_ = *L* + *R*_m_). In these cases, the activities of Rubisco and PEPC are kinetically limited by the CO_2_ supply rather than energetically limited by electron transport, such that $\Gamma$ is insensitive to light intensity. In contrast, the $\Gamma$ values of C_3_–C_4_ plants decrease curvilinearly in response to increasing light intensities (Brown and Morgan 1980; Holaday et al. 1982; Hattersley et al. 1986; Rajendrudu et al. 1986; Cheng et al. 1989; Ku et al. 1991; Dai et al. 1996). The oxygen and temperature responses of the $\Gamma$ values of C_3_–C_4_ plants also appear to be sensitive to light intensity, with higher light intensities associated with higher break-points in each response (Keck and Ogren 1976; Quebedeaux and Chollet 1977; Apel 1980; Morgan and Brown 1980; Brown and Morgan 1980; Holaday et al. 1982, 1984; Hunt et al. 1987; Moore et al. 1987a; Ku et al. 1991; Dai et al. 1996; Vogan et al. 2007). To date, it has not been clear what mechanisms are responsible for the environmental responses of $\Gamma$ in C_3_–C_4_ plants, but mixed states seem likely to be involved (Edwards and Ku 1987; Rawsthorne et al. 1992; Rawsthorne 1992; Leegood and von Caemmerer 1994; von Caemmerer 2000).

**Fig. 3:**
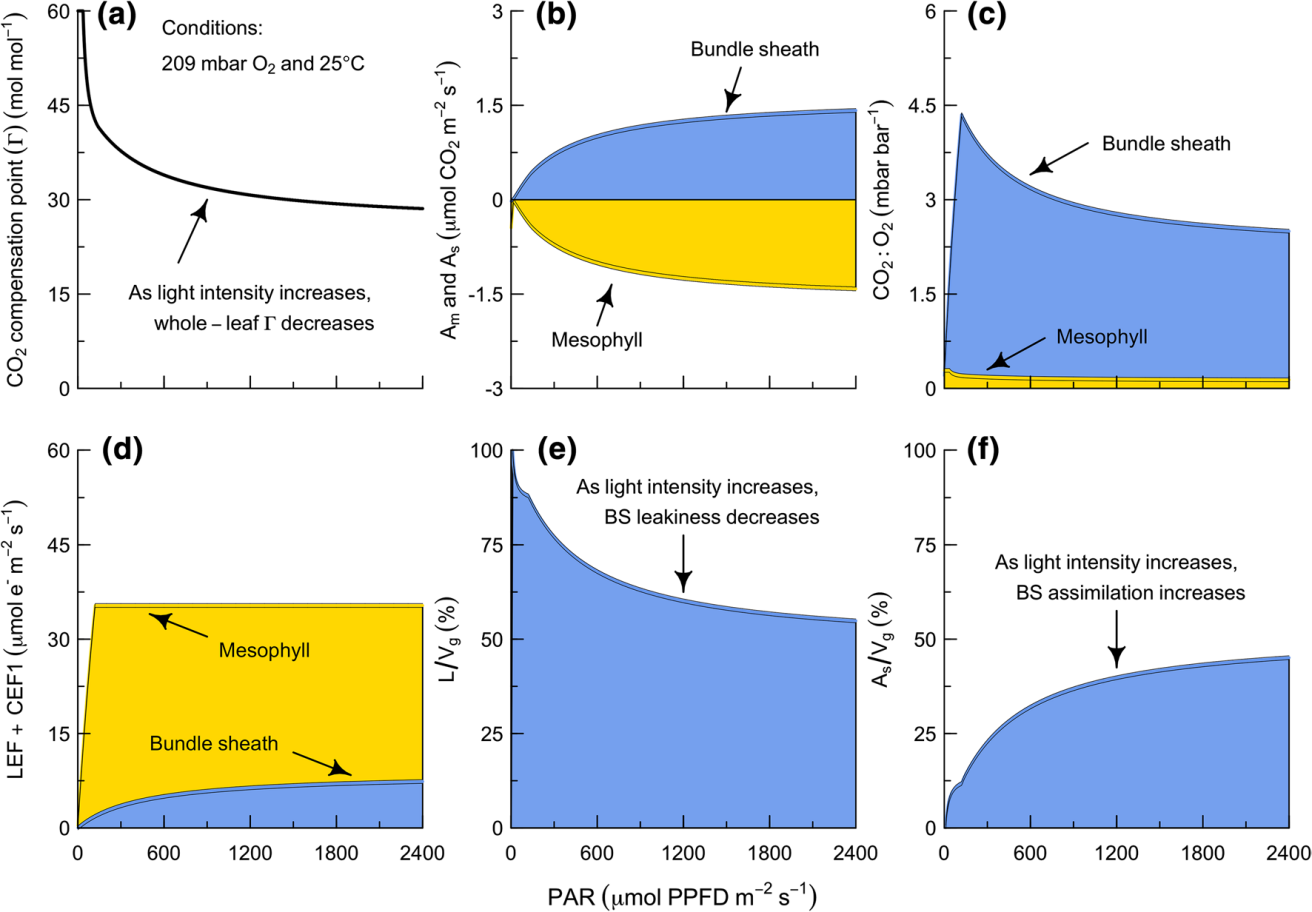
Photosynthetic control of Cyt b_6_f occurs independently in the mesophyll and bundle sheath. To simulate the light dependence of the CO_2_ compensation point ($\Gamma )$ in the Type I C_3_–C_4_ intermediate pathway, the model must be solved in a way that permits each cell type to transition independently between the limiting states. See text for details of each panel. N.B., x-axes are the same for all panels. *A*_m_ and *A*_s_, net rates of CO_2_ assimilation in mesophyll and bundle sheath. *LEF* linear electron flow, *CEF1* cyclic electron flow around PS I, *L* rate of CO_2_ leak from bundle sheath via diffusion, *V*_*g*_ rate of CO_2_ delivery to bundle sheath via glycine shuttle, *PAR* photosynthetically active radiation

The model predicts that a pure state is responsible for the light-insensitive values of $\Gamma$ in the C_3_ and C_4_ cases (i.e., 47 and 0.5 $\mu$bar CO_2_ at 25 °C, respectively), and that a mixed state is responsible for the light-sensitivity of $\Gamma$ in the Type I C_3_–C_4_ case (Fig. 3a). Here, $\Gamma$ corresponds to a state where the CO_2_ uptake driven by Rubisco carboxylase activity in the mesophyll is equivalent to the CO_2_ loss driven by the diffusive leak out of the bundle sheath (i.e., from Eq. 5, *V*_cm_ = *L* + *R*_m_). At this point, the mesophyll is a net CO_2_ source and the bundle sheath is a net CO_2_ sink (Fig. 3b). The mesophyll is a net CO_2_ source because the glycine shuttle is mobilizing fixed carbon from the mesophyll and delivering the derived CO_2_ to the bundle sheath. The bundle sheath is a net CO_2_ sink because the bundle sheath CO_2_ is raised to a level that permits net export of reduced carbon from the bundle sheath Rubisco population. When the mesophyll and bundle sheath are both light-limited, increasing light stimulates CO_2_ delivery to and uptake within the bundle sheath. However, the CO_2_ delivery to the bundle sheath increases faster than the CO_2_ uptake within the bundle sheath, such that the CO_2_:O_2_ ratio rises (Fig. 3c). Since this also increases the rate at which CO_2_ leaks out of the bundle sheath, $\Gamma$ would stabilize if the mesophyll and bundle sheath were both to remain light-limited. Instead, the CO_2_ supply to the bundle sheath stabilizes when the mesophyll becomes light-saturated (Fig. 3d). While the bundle sheath remains light-limited, the activity of Rubisco continues to be stimulated by light, drawing down the CO_2_:O_2_ ratio in the bundle sheath and slowing the rate at which CO_2_ leaks out of the bundle sheath (Fig. 3e). The net effect of these interactions is that a larger fraction of the CO_2_ delivered to the bundle sheath is assimilated, such that $\Gamma$ continues to decrease with light (Fig. 3f). Based on this analysis, we conclude that the light-sensitivity of $\Gamma$ is most likely an indicator of a mixed state. Mixed states are likely to be a key component of C_3_–C_4_ photosynthesis as well as C_4_ photosynthesis. They may be encountered either when leaves are exposed to environmental conditions that are outside those they have acclimated to during growth (e.g., such as $\Gamma$), or under normal growth conditions (e.g., during parts of the diel cycle).

### Basis of temperature sensitivities

While the Cyt b_6_f-based description of electron transport and the mixed state solution permit the model to simulate the response of photosynthesis to variation in light, CO_2_, and O_2_ under constant temperature, additional expressions are required to explain the temperature-dependence of photosynthesis. At present, several different functions are in use and many species-specific parameter sets have been derived for each of them (e.g., see Berry and Raison 1981; Sellers et al. 1996b; von Caemmerer 2000; von Caemmerer et al. 2009; Bernacchi et al. 2013). In principle, any of these expressions can be applied to this model. However, the fundamental challenge in choosing among these functions is neither the appropriate functional form nor the correct parameter values, but the assignment of scaling factors to the correct underlying mechanisms. In particular, it is not yet clear how the intrinsic temperature sensitivities of the electron transport system interact with the intrinsic temperature sensitivities of carbon metabolism. The primary observations that are relevant to this question are: (i) LEF usually exhibits a thermal optimum and declines at high temperatures (e.g., Yamori et al. 2014); (ii) CEF1 is usually stimulated relative to linear electron transport at high temperatures (e.g., Ivanov et al. 2017); (iii) NPQ tends to be stimulated at high temperatures (e.g., Demmig-Adams et al. 2014); (iv) the plastoquinone pool tends to become more oxidized at high temperatures (e.g., Sharkey and Zhang 2010); and (v) the turnover constant of Cyt b_6_f tends to increase with temperature (e.g., Tikhonov 2018). However, it has been difficult to understand the basis of these effects due to the close coordination between the activity of the electron transport system and of carbon metabolism. It has been proposed both that the activity of Rubisco is downregulated at high temperatures due to a limitation on the activity of the electron transport system, and that the activity of the electron transport system is downregulated at high temperatures due to a limitation on the activity of Rubisco.

**Fig. 4:**
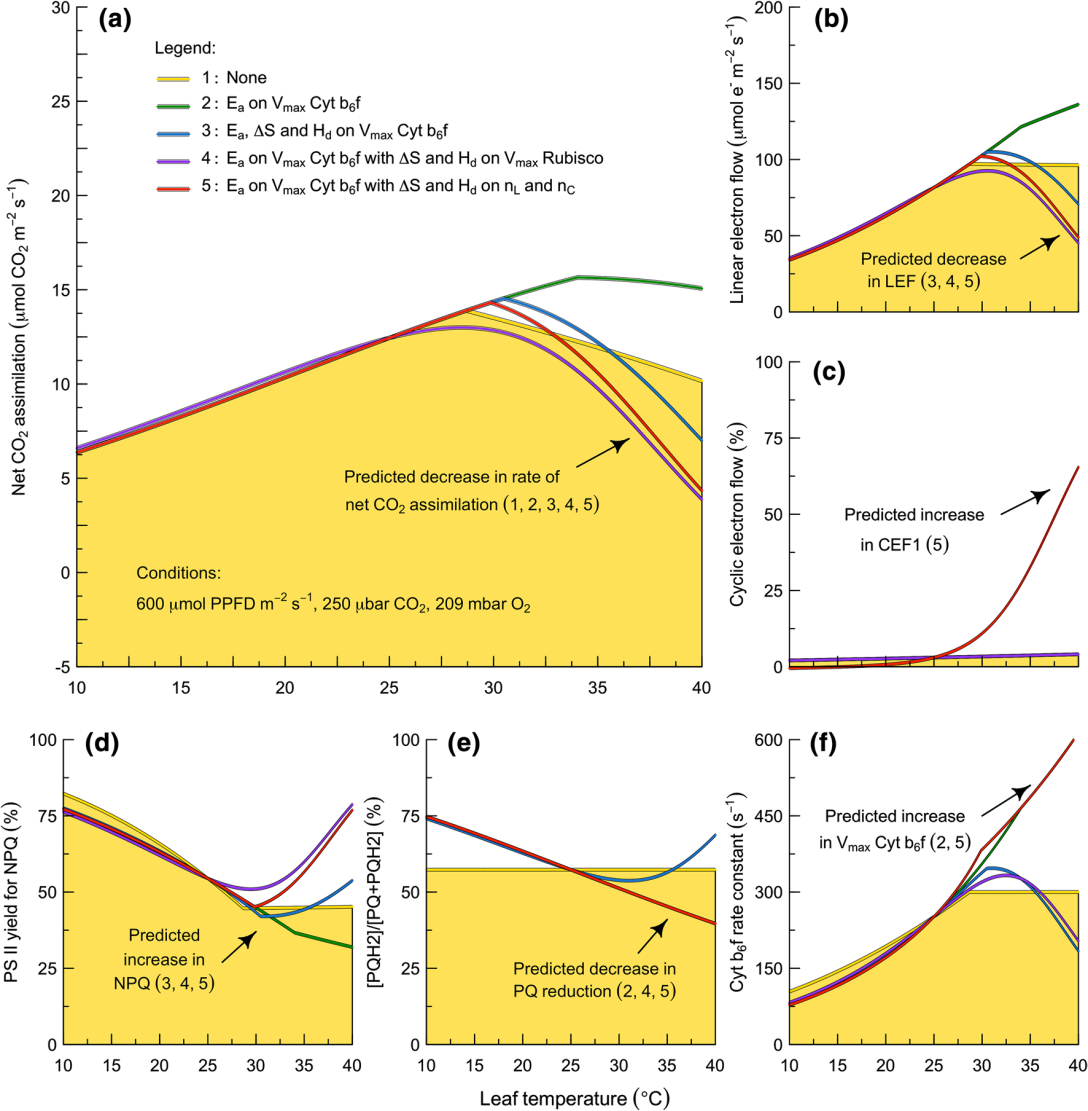
The contributions of Cyt b_6_f and the coupling efficiency to the temperature response of photosynthesis. These simulations examine C_3_ photosynthesis. Yellow shading indicates the reference where the temperature response is driven by carbon metabolism alone. The fifth simulation captures all of the physiological responses that are typically observed. See text for details. N.B., x-axes are the same for all panels. *E*_*a*_ enthalpy of activation (37 kJ mol^−1^), *H*_*d*_ enthalpy of deactivation (220 kJ mol^−1^), *∆S* entropy factor (0.710 kJ mol^−1^ K^−1^). *LEF* linear electron flow, *CEF1* cyclic electron flow around PS I, *NPQ* non-photochemical quenching, *PQ* plastoquinone, *PQH*_*2*_ plastoquinol

To compare the various alternatives quantitatively, we have parameterized the new model with different combinations of the temperature sensitivities that Farquhar et al. (1980a) originally assigned to electron transport through the empirical parameter *J*_max_ (Fig. 4). For all of the simulations, we have maintained a fixed set of temperature responses for carbon metabolism (i.e., following Farquhar et al. 1980a as reviewed by von Caemmerer et al. 2009). Within this background, we have selectively varied the localization of the activation and deactivation terms from *J*_max_ (*E*_a_ of 37 kJ mol^−1^, *H*_d_ 220 kJ mol^−1^, Δ*S* 0.710 kJ mol^−1^ K^−1^). The alternative localizations are: (1) no assignment of the *J*_max_ coefficients; (2) *J*_max_ activation term on *V*_max_ of Cyt b_6_f; (3) *J*_max_ activation and deactivation terms on *V*_max_ of Cyt b_6_f; (4) *J*_max_ activation term on *V*_max_ of Cyt b_6_f and deactivation terms on *V*_max_ of Rubisco; (5) *J*_max_ activation term on *V*_max_ of Cyt b_6_f and deactivation terms on the efficiency of coupling between electron transport and ATP production. While all five parameterizations predict a thermal optimum in net CO_2_ assimilation (Fig. 4a), each predicts a distinct combination of temperature responses in the rate of LEF (Fig. 4b), the rate of CEF1 (Fig. 4c), the level of NPQ of PS II (Fig. 4d), the redox poise of the plastoquinone pool (Fig. 4e), and the turnover constant of Cyt b_6_f (Fig. 4f). The predictions only capture all of the primary features of the observations when the activation term from *J*_max_ is assigned to *V*_max_ of Cyt b_6_f and the deactivation term from *J*_max_ is assigned to the *n*_L_ and *n*_*C*_ parameters that describe the efficiency of coupling between NADPH, Fd, and ATP production (Fig. 4b–f; red lines). This suggests that increasing temperatures may have two general effects on the electron transport system: first, increasing the *V*_max_ of Cyt b_6_f; and second, decreasing the efficiency of coupling between NADPH, Fd, and ATP production.

These results have three main implications. First, this modeling framework provides a new foundation for understanding and predicting the temperature responses of photosynthesis. In this analysis, we have leveraged existing temperature response functions to illustrate the model’s ability to simulate multiple observable quantities derived from gas-exchange, fluorescence, and absorbance measurements. However, this application should not be interpreted as an endorsement either of this specific functional form or this specific parameterization. Further research is needed to evaluate the most appropriate functional form and parameterization of the temperature responses. Second, it is likely that increasing temperature stimulates the maximum activity of Cyt b_6_f, but the magnitude of this effect is in need of improved quantification. Although assigning the activation term from *J*_max_ to *V*_max_ of Cyt b_6_f permits a qualitatively realistic simulation of the temperature response, this is not expected to be quantitatively realistic because the *V*_max_ of Cyt b_6_f is not equivalent to the *J*_max_ parameter of Farquhar et al. (1980a). The *J*_max_ parameter represents the rate of electron transport that occurs under saturating light and saturating CO_2_, and in C_3_ leaves at 25 °C this is around one-half of the true maximum potential rate of electron transport that is defined by the substrate-saturated rate of electron flow through Cyt b_6_f (Johnson and Berry 2021). In vivo and in vitro evaluations of the temperature sensitivity of the *V*_max_ of Cyt b_6_f are needed. Third, the reversible decline in photosynthesis at high temperatures may be caused by a decline in the efficiency of coupling between NADPH, Fd, and ATP production. In principle, such a decline in coupling efficiency might be driven by a temperature effect on: (i) the coupling between electron flow and proton translocation at Cyt b_6_f; (ii) the proton “leakiness” of the thylakoid membrane; (iii) the coupling between proton translation and ATP formation at ATP synthase; and/or (iv) other related mechanisms. This possibility is distinct from the alternative hypotheses that are currently dominant, and the full range of hypotheses deserve careful experimental evaluation. In the next section, we will illustrate how the model can be used as a tool to aid in this type of quantitative interpretation of physiological measurements.

## Model applications

This model can be used for forward simulations and for inverse fitting. In this section, we provide examples of both types of applications. We first present forward simulations that illustrate the responses of C_3_, Type I C_3_–C_4_, and C_4_ photosynthesis to light, carbon dioxide, and temperature. The simulations demonstrate how the Cyt b_6_f-based expressions for electron transport, the hierarchical solution for mixed states, and the temperature dependencies come together in the overall performance of the model. We then present inverse analyses that illustrate how the model can be used to interpret gas-exchange measurements of a Type I C_3_–C_4_ plant, *Flaveria* *chloraefolia*. The inverse analyses demonstrate that population-level variation in the CO_2_ compensation point in this species can be explained by variable allocation of photosynthetic capacity to the bundle sheath. We conclude by discussing key questions that are raised by this framework and posing a novel hypothesis about the origins of C_4_ photosynthesis.

### Forward simulations

**Fig. 5:**
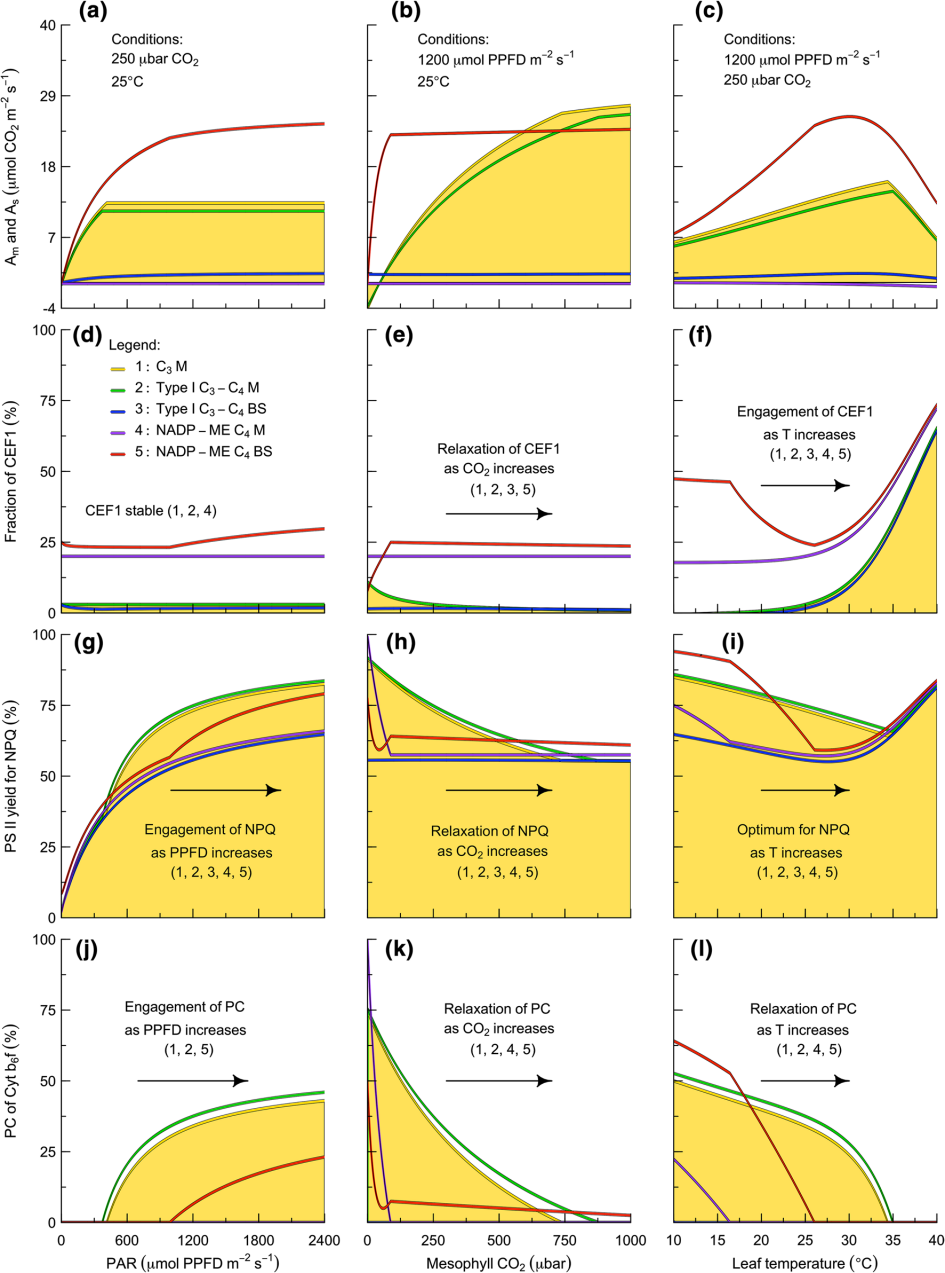
Responses of C_3_, Type I C_3_–C_4_, and C_4_ photosynthesis to light, carbon dioxide, and temperature. For each environmental variable, simulations are plotted separately for C_3_ mesophyll, Type I C_3_–C_4_ mesophyll and bundle sheath, and NADP-ME C_4_ mesophyll and bundle sheath. In each plot, yellow shading indicates the reference simulation that corresponds to the C_3_ case. Parameters are as in Table 2. See text for other details. N.B., x-axes are the same for panels in each column. *LEF* linear electron flow, *CEF1* cyclic electron flow around PS I, *NPQ* non-photochemical quenching of PS II, *PC* photosynthetic control of Cyt b_6_f, *M* mesophyll, *BS* bundle sheath, *PAR* photosynthetically active radiation

The responses of photosynthesis to light, carbon dioxide, and temperature vary substantially between the C_3_, Type I C_3_–C_4_, and C_4_ pathways (Fig. 5a–c). Part of the variation is controlled by pathway- and cell-type-specific differences in photosynthetic capacity which control the absolute amounts of Fd, NADPH, and ATP produced and consumed (Table 2). The remainder of the variation is controlled by pathway- and cell-type-specific differences in photosynthetic metabolism which control the relative amounts of Fd, NADPH, and ATP that are produced and consumed (Appendix I: Electronic Supplementary Material). In this model, three forms of regulation coordinate the energy supply from electron transport with the energy demand from carbon metabolism. In any given pathway and cell type, the flux through LEF versus CEF1 is dynamically modulated to establish a sink-appropriate balance between the supply of ATP and the supply of Fd and NADPH (Fig. 5d–f). The partitioning between LEF and CEF1 is controlled by the distribution of excitation between PS I and PS II. This depends on the relative absorption cross-section of each population of photosynthetic units and the level of connectivity between photosynthetic units, and is dynamically modulated through NPQ (Fig. 5g–i). Once the energy supply and demand are balanced in relative terms, the final requirement is ensuring that they are also balanced in absolute terms. This is achieved via PC of Cyt b_6_f, which restricts the rate of electron flow to the capacity of carbon metabolism to provide acceptors (Fig. 5j–l). Across the three pathways, PC tends to be relaxed at low light and to become engaged as the light intensity is increased (Fig. 5j), to be engaged at low CO_2_ and to relax as the CO_2_ concentration is increased (Fig. 5k), and to be engaged at low temperature and to relax as the temperature is increased (Fig. 5l). However, the Type I C_3_–C_4_ bundle sheath remains light-limited under all of these conditions, and the C_4_ mesophyll remains light-limited under most conditions except the lowest CO_2_ concentrations and lowest temperatures. As a result, mixed states are common in both the Type I C_3_–C_4_ and C_4_ pathways along all three environmental axes.

These simulations demonstrate that this framework both reproduces the core dynamics of earlier photosynthesis models and introduces new capabilities with respect to the interactions between the light, CO_2_, and temperature responses. To date, the expressions for light-saturated photosynthesis that were summarized by von Caemmerer (2000) have been successful at reproducing: (i) a CO_2_ compensation point for C_3_–C_4_ plants that is intermediate to those of C_3_ and C_4_ plants at atmospheric O_2_ levels; (ii) a non-linear response of the CO_2_ compensation point to variation in O_2_ in C_3_–C_4_ plants; and (iii) a curvilinear response of net CO_2_ assimilation to variation in CO_2_ in C_3_ and C_3_–C_4_ plants, as well as a biphasic response in C_4_ plants. The framework we have described here builds on this foundation by introducing expressions for light-limited photosynthesis that are successful at reproducing: (iv) a curvilinear response of net CO_2_ assimilation to variation in light in C_3_, C_3_–C_4_, and C_4_ plants; (v) a CO_2_ compensation point that is light-dependent in C_3_–C_4_ plants; and (vi) a light-dependent decline in net CO_2_ assimilation at high temperatures in C_3_, C_3_–C_4_, and C_4_ plants. Compared to the empirical expressions that are currently used to describe the light response, this more mechanistic approach has two main advantages: it provides a clearer interpretation of how photosynthesis works as an interactive system, and it provides a stronger connection to quantities that are directly observable. For example, the model enables analysis of the flux of light available to the mesophyll vs. bundle sheath (i.e., a function of the absorptance of each chloroplast population), and the light saturation point of the mesophyll vs. bundle sheath (i.e., a function of the balance between the Cyt b_6_f and Rubisco in each chloroplast population). These limits are quite important because they structure the suite of regulatory interactions that coordinate electron transport with carbon metabolism (e.g., Fig. 5). However, at present there is relatively little quantitative understanding of how pigment and protein distributions vary within and between C_3_, Type I C_3_–C_4_, and C_4_ plants. In the next section, we turn to inverse fitting to explore pigment and protein distributions and their functional consequences.

### Inverse fitting

By fitting the model to observations within an inversion framework, the total amounts of pigment and protein as well as the relative allocation of pigment and protein to the bundle sheath can be inferred rather than prescribed. As an example of this approach, we have applied the model to the interpretation of the photosynthetic performance of *Flaveria* *chloraefolia* (Asteraceae). *F.* *chloraefolia* is a Type I C_3_–C_4_ species that has been grown and studied under controlled conditions for decades (e.g., Powell 1978; Holaday et al. 1984; Holaday and Chollet 1984; Reed and Chollet 1985; Bauwe and Chollet 1986; Edwards and Ku 1987; Chastain and Chollet 1989; Ku et al. 1991; Dai et al. 1996; Kopriva et al. 1996; Pfündel and Pfeffer 1997; Leonardos and Grodzinski 2000, 2003; Engelmann et al. 2003; Huxman and Monson 2003; Westhoff and Gowik 2004; McKown et al. 2005; McKown and Dengler 2007; Kocacinar et al. 2008; Vogan and Sage 2011; Schulze et al. 2013; Aldous et al. 2014; Mallmann et al. 2014; Way et al. 2014; Stata et al. 2016; Lyu et al. 2021). However, there are very few studies of this species under field conditions (e.g., Van Auken et al. 2007; Peralta-García et al. 2016; Ochoterena et al. 2020; Peralta-García et al. 2020; Pisanty et al. 2020; Rodríguez-Sánchez et al. 2020). We compared the photosynthetic performance of a greenhouse-grown research population at the Carnegie Institution in Stanford, CA to that of naturally-occurring populations at the Blue Hole Ciénega in Guadalupe County, NM (34° 56ʹ 8ʺ N, 104° 40ʹ 30ʺ W), and the Diamond Y Spring Preserve in Pecos County, TX (31° 0ʹ 36ʺ N, 102° 55ʹ 5ʺ W). Measurements of the CO_2_ response of mature leaves were made on the research population in CA during the mid-winter, and on the wild populations in NM and TX during the mid-summer. At each site, measurements were made for *n* = 7–15 individual leaves. For each leaf, a LI-6400XT (LI-COR, Lincoln, NE) system was used to vary CO_2_ from 0 to 1000 $\mu$bar, at 210 mbar O_2_, with a light intensity of 1500 $\mu$mol PPFD m^−2^ s^−1^ and leaf temperatures ranging between 28 and 33 °C.

**Fig. 6:**
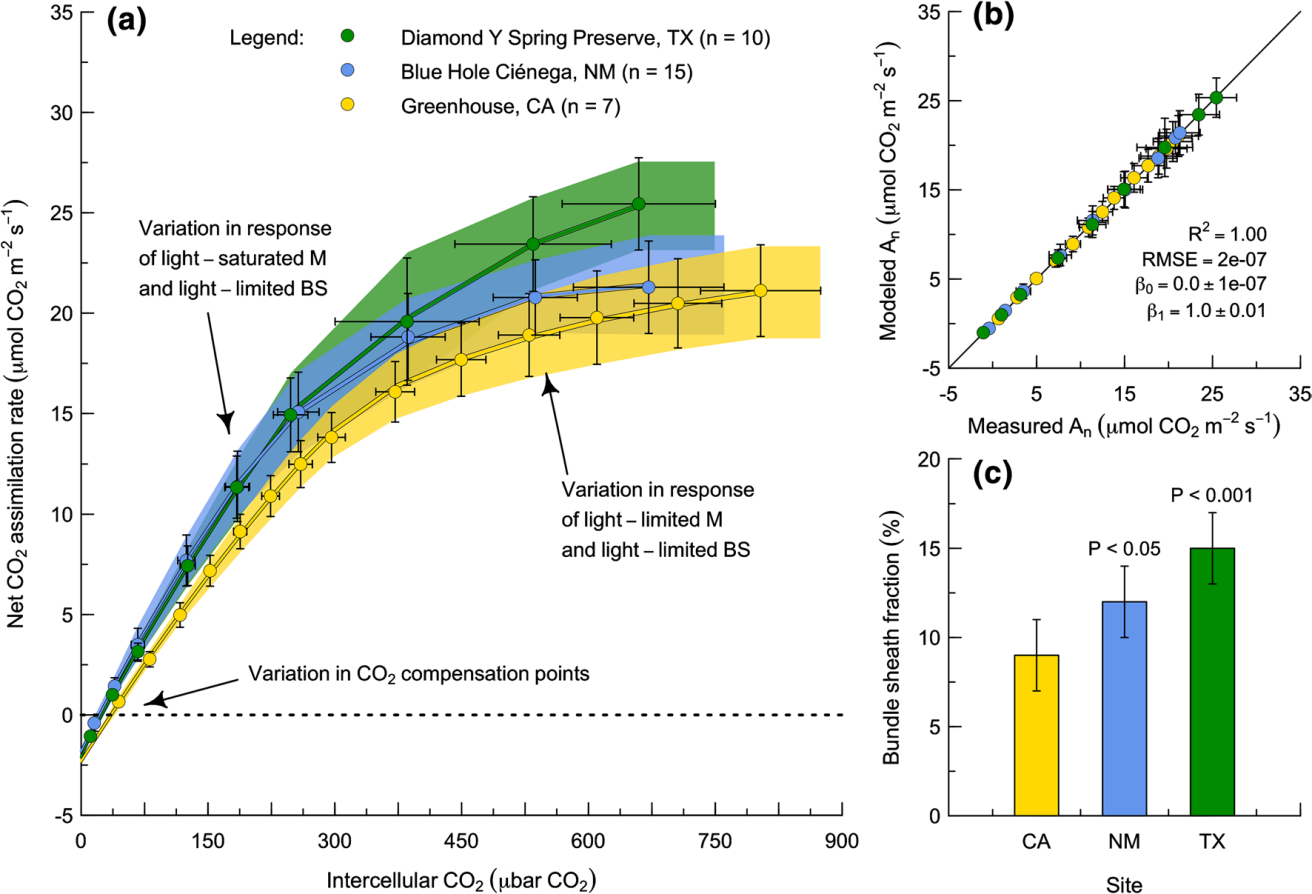
Patterns and determinants of photosynthetic performance in *Flaveria* *chloraefolia* (Type I C_3_–C_4_) at three sites in different environments. **a** At each site, the CO_2_ response of photosynthesis was assayed between 0 and 1000 $\mu$bar CO_2_ at 1500 $\mu$mol PPFD m^−2^ s^−1^, 27.5 to 32.5 °C, and 210 mbar O_2_ using a LI-6400XT (LI-COR, Lincoln, NE) (points). The photosynthesis model was then fit to each individual CO_2_ response (lines). **b** The model fit the measurements well, without any bias and with little noise. **c** There was significant variation between sites in the fraction of total absorptance. *V*_max_ of Cyt b_6_f, and *V*_max_ of Rubisco in the bundle sheath. See text for details

To interpret the basis of the measured responses, we fit each individual CO_2_ response curve to the model using an optimization framework (Fig. 6). Tests with synthetic data that mimicked the real sampling design and error characteristics demonstrated that the fitting procedure could be expected to retrieve up to four free parameters to within ± 1% of their true values. The four free variables we selected for fitting were: (i) mesophyll conductance to CO_2_, (ii) *V*_max_ of Cyt b_6_f, (iii) *V*_max_ of Rubisco, and (iv) the fraction of the total absorptance, *V*_max_ of Cyt b_6_f and *V*_max_ of Rubisco in the bundle sheath. In the measurements, there was leaf-to-leaf variation in three aspects of the CO_2_ response: (i) the sensitivity of net CO_2_ assimilation to CO_2_ at high intercellular CO_2_; (ii) the sensitivity of net CO_2_ assimilation to CO_2_ at low intercellular CO_2_; and (iii) the CO_2_ compensation point (Fig. 6a; points). The largest differences were between the greenhouse-grown research population in CA and the two wild populations in NM and TX. Compared to the greenhouse-grown/laboratory-measured population, the naturally-occurring/field-measured populations tended to exhibit higher sensitivities of net CO_2_ assimilation to CO_2_ across the entire sampled range as well as lower CO_2_ compensation points. The model was able to fit these patterns by varying the mesophyll conductance to CO_2_, the total amounts of Cyt b_6_f and Rubisco, and the fractional allocations of the total absorptance, *V*_max_ of Cyt b_6_f and *V*_max_ of Rubisco to the bundle sheath (Fig. 6a; lines and shaded confidence intervals). The fits did not exhibit any systematic biases and had relatively little noise (Fig. 6b). Within each of the three populations, there was some leaf-to-leaf variation in mesophyll conductance to CO_2_, *V*_max_ of Cyt b_6_f, and *V*_max_ of Rubisco (Table 3). However, the parameter that exhibited the largest systematic variation between the three populations was the bundle sheath fraction of absorptance, Cyt b_6_f, and Rubisco (i.e., with a range from 7 to 17%; Fig. 6c; Table 3).

**Table 3:** Parameter estimates for *Flaveria* *chloraefolia* (Type I C_3_–C_4_) in CA, NM, and TX

Parameters	Parameter estimates: 50th (25th, 75th)			Pairwise comparisons		
Description	CA	NM	TX	CA:NM	CA:TX	NM:TX
*g*_*m*_ (mol CO_2_ m^−2^ s^−1^)	0.28 (0.19, 0.36)	0.40 (0.33, 0.48)	0.35 (0.22, 0.47)	*P* > 0*.*050	*P* > 0*.*050	*P* > 0*.*050
*V*_*max*_ Cyt b_6_f ($\mu$mol e- m^−2^ s^−1^)	117 (98, 137)	133 (120, 146)	193 (146, 240)	*P* > 0*.*050	*P* < 0*.*001	*P* = 0*.*001
*V*_*max*_ Rubisco ($\mu$mol CO_2_ m^−2^ s^−1^)	52 (41, 63)	61 (55, 67)	59 (51, 67)	*P* > 0*.*050	*P* > 0*.*050	*P* > 0*.*050
Bundle sheath allocation (%)	9 (7, 11)	12 (10, 14)	15 (13, 17)	*P* = 0*.*044	*P* < 0*.*001	*P* = 0*.*018

The fitting procedure estimated four free variables. Mesophyll conductance to CO_2_ was treated as a temperature-invariant parameter. The maximum activities for Cyt b_6_f and Rubisco were scaled from the measurement temperatures to a reference temperature of 25 °C. The bundle sheath allocation was treated as a single parameter representing fractional allocation of absorptance, Cyt b_6_f, and Rubisco to the bundle sheath. Pairwise comparisons were performed using *t* tests

These results have three notable features. First, the quality-of-fit statistics indicate that the model structure is formulated in a way that captures the major features of the measurements. This is significant because it suggests that model parameterization, rather than model structure, could be the primary factor contributing to the long-standing discrepancy between the predicted versus observed performance of Type I C_3_–C_4_ plants (i.e., why they have not exhibited higher rates of net CO_2_ assimilation than C_3_ plants under saturating light, moderate leaf temperatures, and modern atmospheric CO_2_ and O_2_ levels; as discussed in the Introduction). Specifically, in previous applications of the modeling framework described by von Caemmerer (2000), the parameterizations of C_3_ and Type I C_3_–C_4_ simulations have assumed equivalent photosynthetic capacities. This may be unrealistic, and leads to the second point: the ranges of variation in the estimated values of mesophyll conductance to CO_2_, *V*_max_ of Cyt b_6_f, and *V*_max_ of Rubisco are limited. The restricted ranges could reflect underlying anatomical constraints which facilitate the operation of the glycine shuttle, such as contact between mesophyll and bundle sheath cells. This is significant because it indicates that realizing the biochemical advantages of the glycine shuttle may entail an anatomical trade-off, and such a trade-off could help to reconcile observations of Type I C_3_–C_4_ physiology with current understanding of the glycine shuttle. For example, if C_3_–C_4_ plants are restricted to a limited range of specific leaf areas, this could limit the range of V_max_ values of Cyt b_6_f and Rubisco below that of C_3_ plants and help to explain lower area-based measurements of the rate of net CO_2_ assimilation. Third, the bundle sheath fraction of absorptance, Cyt b_6_f, and Rubisco not only varies significantly between populations, but also is consistently higher in the wild populations in NM and TX than in the greenhouse-grown population in CA. This indicates that the allocation of photosynthetic capacity to the bundle sheath is flexible, and suggests that C_3_–C_4_ intermediate plants optimize pigment and protein distributions to maximize the benefits and minimize the costs of glycine shuttling in different environments. In combination, these findings demonstrate the potential of the inversion-based approach to the interpretation of physiological measurements. Developing this approach further requires revisiting the quantitative definition of the energetics of glycine shuttling. This is the topic we turn to in the final section.

### Key questions

The structure of the Type I C_3_–C_4_ model we have implemented here is a quantitative expression of a conceptual model in which the confinement of glycine decarboxylation to the bundle sheath has only one functional consequence: under conditions that promote oxygen fixation by mesophyll Rubisco, glycine is transported into the bundle sheath and the CO_2_ released from bundle sheath GDC activity builds up, increasing the bundle sheath CO_2_/O_2_ ratio and increasing the efficiency of CO_2_ fixation by the bundle sheath Rubisco. Several independent lines of evidence support the inference that confinement of GDC activity to the bundle sheath drives diffusive transport of glycine into the bundle sheath (Rawsthorne and Hylton 1991; Leegood and von Caemmerer 1994) and increases the efficiency of CO_2_ fixation by the bundle sheath Rubisco (Moore et al. 1987b; Keerberg et al. 2014). However, the activity of GDC is coordinated with the activity of serine hydroxymethyl transferase such that the overall products of glycine decarboxylation include serine, NH_3_, and NADH, in addition to CO_2_ (Woo and Osmond 1976; Sarojini and Oliver 1983). While this implies that serine, NH_3_, NADH, and/or stoichiometrically equivalent products of their metabolism must return from the bundle sheath to the mesophyll, it is not yet clear which mechanisms maintain carbon, nitrogen, and redox balance (Rawsthorne et al. 1988a, 1992; Leegood and von Caemmerer 1994; Mallmann et al. 2014; Schlüter et al. 2017).

Since NH_3_ can escape to the atmosphere (Farquhar et al. 1980b; Johnson and Berry 2013), it seems likely that it is reassimilated in the bundle sheath via glutamine synthetase and/or glutamate synthase (Rawsthorne et al. 1988b, 1992; Monson and Rawsthorne 2000). If this occurs, then the glycine shuttle could either: (i) create an additional ATP demand in the bundle sheath (i.e., NH_3_ reassimilated in bundle sheath chloroplasts by glutamine synthetase alone); and/or (ii) create an additional ATP, NADPH, and Fd demand in the bundle sheath (i.e., NH_3_ reassimilated in bundle sheath chloroplasts by glutamine synthetase and glutamate synthase). In theory, these energetic demands could potentially be satisfied by several different energy-balancing mechanisms: (iii) a shift in the balance of linear and cyclic electron flow within the bundle sheath chloroplasts; (iv) a shift in the balance of NADH oxidation via the malate valve, cytochrome oxidase, and alternative oxidase pathways within the bundle sheath mitochondria; and/or (v) shuttling of 3-phosphoglyceric acid and dihydroxyacetone phosphate between the bundle sheath and mesophyll. Considering the range of candidate mechanisms from a stoichiometric perspective indicates that there is potential for interactions between the fate of NH_3_ and the fate of NADH within the glycine shuttle, and also for interactions between the glycine, aspartate, and malate shuttles (e.g., Mallmann et al. 2014; Bellasio 2017; Schlüter and Weber 2020). However, it is not yet clear which of these interactions actually play out.

**Fig. 7:**
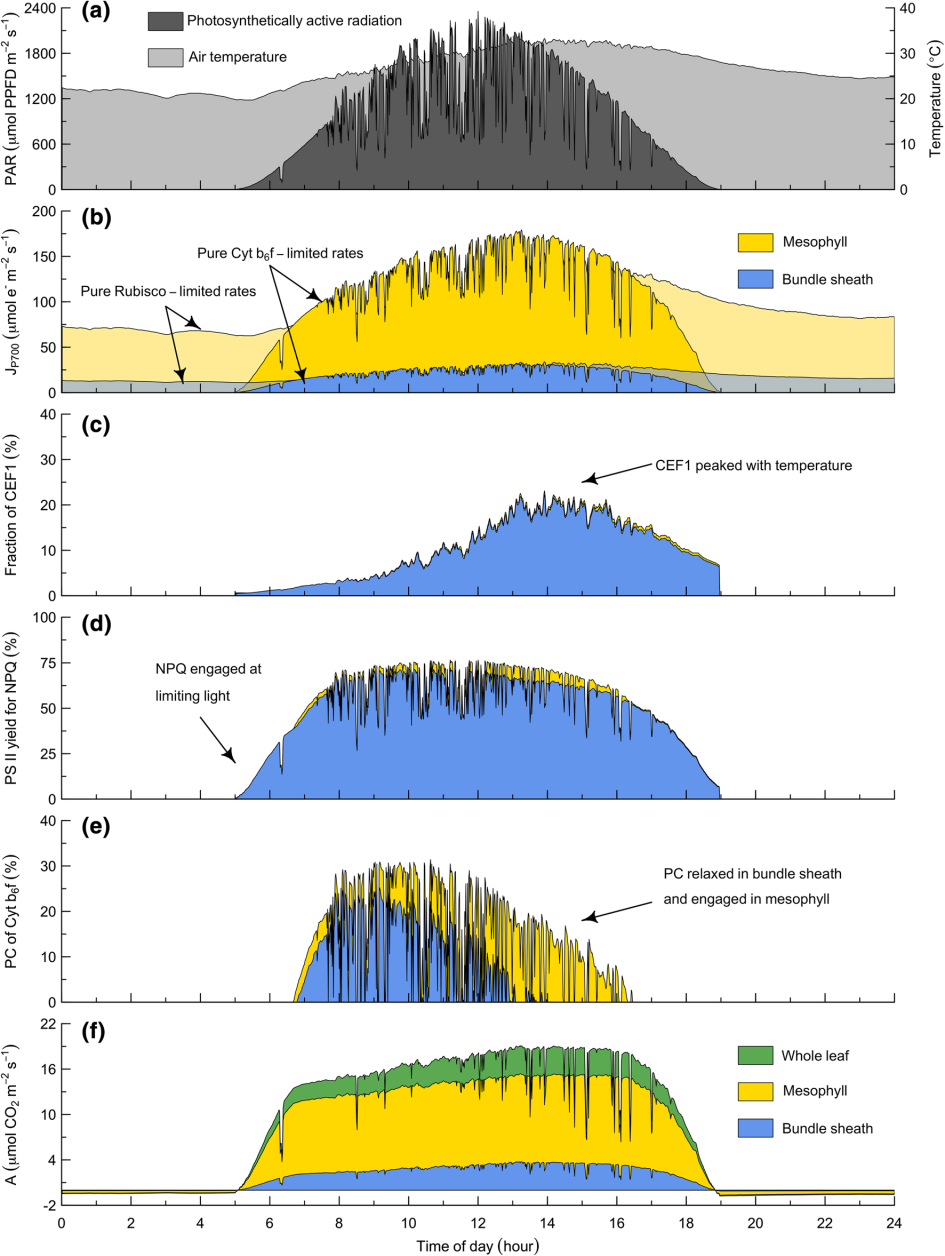
Model simulation of the photosynthetic performance of *Flaveria* *chloraefolia* (Type I C_3_–C_4_) over the diel cycle at the Diamond Y Spring Preserve. The photosynthesis model was parameterized with the fitted values of the physiological variables from the Texas site, and then driven with measurements of top-of-canopy irradiance and air temperature from that site over one day in July. See text for details of methods and discussion of results. N.B., *x*-axes are the same for all panels. *CEF1* cyclic electron flow around PS I, *NPQ* non-photochemical quenching of PS II, *PC* photosynthetic control of Cyt b_6_f, *PAR* photosynthetically active radiation

To explore the ecological context for these biochemical interactions, we parameterized the photosynthesis model with the fitted values of the physiological variables from the Diamond Y Spring Preserve (Table 3) and then simulated Type I C_3_–C_4_ photosynthesis at this site over one day in July (Fig. 7). For simplicity, the leaf temperature was prescribed as equivalent to air temperature, and mesophyll CO_2_ and O_2_ were prescribed as 250 $\mu$bar and 210 mbar, respectively (i.e., omitting the dynamic coupling between photosynthesis, stomatal conductance, and the energy balance). All other input parameters were as given in Table 2. With this approach, the diel cycle of light and temperature (Fig. 7a) determines the limits of the potential rates of electron transport (Fig. 7b). From these limits, CEF1 (Fig. 7c), NPQ of PS II (Fig. 7d), and PC of Cyt b_6_f (Fig. 7e) then regulate the actual linear electron flow to a rate that remains coordinated with the capacity of carbon metabolism to provide electron acceptors (Fig. 7f). These dynamics illustrate both challenges and opportunities for bundle sheath assimilation of NH_3_ via glutamine synthetase and/or glutamate synthase. In particular, the small fractional allocation of photosynthetic capacity to the bundle sheath implies that reassimilation of mesophyll-derived NH_3_ would be likely to dominate the bundle sheath energy budget. While the increased ATP to Fd and NADPH ratio associated with glutamine synthetase activity could potentially be met through increased CEF1, it could also be limited by decreased coupling efficiency at high temperatures. Alternatively, a decreased coupling efficiency might permit the chloroplast chain to support the increased Fd and NADPH to ATP ratio associated with glutamate synthase activity. In either case, the bundle sheath would be likely to make the most efficient use of the available light under conditions where state transitions optimize the absorption cross-sections of PS I and PS II (i.e., without wasting absorbed light through the heat-dissipating forms of non-photochemical quenching) such that electrons flow to the sink at the maximum possible rate (i.e., without inducing PC of Cyt b_6_f).

In this context, it is intriguing to consider whether the malate and aspartate shuttles might have originally functioned to smooth out the energy supply and demand associated with the glycine shuttle, and thereby to maintain the bundle sheath in an energetically balanced state. If this is the case, it would imply that the CO_2_-concentrating function of the malate and aspartate shuttles was not the reason for their origin (i.e., much like the spandrels of San Marco; Gould and Lewontin 1979). Such a hypothesis can be explored quantitatively if the chloroplast and mitochondrial electron transport chains are conceptualized as part of a single, interactive system that balances energy supply and demand, subject to the inherent kinetic limits of each chain and the manner in which they are regulated. The model we have described here provides a framework for performing this type of analysis and determining which combinations of potential interactions actually operate in vivo in different plants and under different environmental conditions. Since *Flaveria* *chloraefolia* co-exists with C_3_ and C_4_ competitors at the Diamond Y Spring and the Blue Hole Ciénega, further study of these communities may provide insight into the mechanisms of competitive coexistence and whether the ecological conditions here allow the Type I C_3_–C_4_ pathway to represent an evolutionarily stable strategy. In a global change context, such understanding may have particularly important applications to conservation of rare, threatened, and endangered species (e.g., Pisanty et al. 2020) and engineering of the C_4_ pathway into C_3_ crops (e.g., Ermakova et al. 2020).

## Conclusions

1. We have developed a quantitative model of C_3_, C_3_–C_4_ intermediate, and C_4_ photosynthesis that relates the factors limiting electron transport and carbon metabolism, the regulatory processes that coordinate these metabolic domains, and the overall responses to light, carbon dioxide, and temperature. The model describes the steady-state responses of leaf-level photosynthesis to these environmental factors within the ranges of conditions that leaves have acclimated to during growth.
2. This framework has three unique features. First, mechanistic expressions describe how the Cytochrome b_6_f complex controls electron transport in mesophyll and bundle sheath chloroplasts. Second, the mesophyll and bundle sheath expressions are coupled in a way that represents how feedback regulation of Cyt b_6_f coordinates electron transport and carbon metabolism. Third, the temperature sensitivity of Cyt b_6_f is differentiated from that of the coupling between NADPH, Fd, and ATP production.
3. Using this framework, we have presented simulations demonstrating that the unique light dependence of the CO_2_ compensation point in C_3_–C_4_ leaves can be explained by co-occurrence of light-saturation in the mesophyll and light-limitation in the bundle sheath. We have also presented inversions demonstrating that population-level variation in the CO_2_ compensation point in a Type I C_3_–C_4_ species, *Flaveria* *chloraefolia*, can be explained by variable allocation of photosynthetic capacity to the bundle sheath.
4. While there are substantial uncertainties about how the glycine shuttle works, this CO_2_-concentrating mechanism is likely to provide advantages in the net CO_2_ assimilation rate and resource-use efficiencies under some combinations of light, CO_2_, and temperature, and disadvantages under others. C_3_–C_4_ intermediate plants may optimize pigment and protein distributions to maximize the benefits and minimize the costs of glycine shuttling under different environmental conditions.
5. Understanding this optimization quantitatively holds promise for explaining why the Type I C_3_–C_4_ pathway occupies such a key place in evolutionary history and yet remains so ecologically rare. It also holds promise for explaining the functional relationships between the glycine, malate, and aspartate shuttles, and evaluating the hypothesis that the C_4_ pathway originally evolved to smooth out energy supply and demand, rather than to concentrate CO_2_.

## Supplementary Information

Below is the link to the electronic supplementary material.

Supplementary file1 (PDF 115 KB)

## Data Availability

The datasets analyzed in this study are available with the code, as described below.

## Code availability

The model described in this manuscript was implemented in MATLAB R2020b. The code is published under an MIT License, available on GitHub (https://github.com/jenjohnson), and archived on Zenodo (10.5281/zenodo.5577145).
