# Supplementary material for: The limiting factors and regulatory processes that control the environmental responses of C3, C3–C4 intermediate, and C4 photosynthesis
Source: Oecologia. 2021 Oct 29;197(4):841–66. doi: 10.1007/s00442-021-05062-y (PMC8591018; doi:10.1007/s00442-021-05062-y)
Supplement: Supplementary file 1 — Supplementary file1 (PDF 115 KB) [file 442_2021_5062_MOESM1_ESM.pdf]

Electronic Supplementary Material (ESM): The limiting factors and regulatory processes that control the environmental responses of C_3, C_3-C_4 intermediate, and C_4 photosynthesis

Jennifer E. Johnson[1] · **Christopher B. Field**[1, 2] · **Joseph A. Berry**[1]

Abstract The expressions in Appendices I-V are given for idealized C_3, Type I C_3-C_4, and NADP-ME C_4 cases. These cases allow us to focus on developing an explicit representation of chloroplast electron transport, while maintaining an implicit representation of mitochondrial electron transport. Appendix I gives expressions that describe the whole-leaf net assimilation rate (A), in relation to net CO_2 assimilation in the mesophyll (A_m) and the bundle sheath (A_s). These expressions are relevant under all environmental conditions. Appendices II, III, and IV give additional expressions that describe the dependence of A_m and A_s on light, pCO_2, and pO_2. Appendix II gives expressions for a pure light-limited state, and Appendix III gives expressions for a pure light-saturated state. Appendix IV gives expressions for mixed light-limited/light-saturated states, and Appendix V gives the solution to the model. For the analyses in this paper, the equations in Appendices I-V were implemented in MATLAB (2020b, The MathWorks, Natick, MA, USA). The Symbolic Math Toolbox was used to generate analytical solutions to the systems of equations specified in Appendix V, and the Global Optimization and Parallel Computing Toolboxes were used to fit the model to data.

[1]Department of Global Ecology, Carnegie Institution for Science, 260 Panama Street, Stanford CA 94305 USA

[2]Woods Institute for the Environment, Stanford University, 473 Via Ortega, Stanford CA 94305 USA

Correspondence: Jennifer E. Johnson, Email: jjohnson@carnegiescience.edu, ORCID ID: 0000-0003-2181-8402

https://doi.org/10.1007/s00442-021-05062-y

Appendix I: Governing rate equations

Steady-state electron transport

We begin by describing a metabolic state in which the supplies of Fd, NADPH, and ATP from chloroplast electron transport are dynamically coordinated with the energetic demands from carbon metabolism. For the C_3 and the Type I C_3-C_4 mesophyll, the rates of electron transport through PS II and PS I depend on the activity of the photosynthetic carbon reduction (PCR) and photosynthetic carbon oxidation (PCO) cycles:

$$J_{P680m} = 4 \cdot V_{cm} + 4 \cdot V_{om} \tag{1a}$$

$$J_{P700m} = (3 \cdot V_{cm} + 3.5 \cdot V_{om} - J_{P680m} \cdot n_L)/n_C + J_{P680m} \tag{1b}$$

where J_{P680m} is the total rate of linear electron flow (LEF) through mesophyll PS II (mol e^- m^{-2} s^{-1}), J_{P700m} is the total rate of LEF and cyclic electron flow (CEF1) through mesophyll PS I (mol e^- m^{-2} s^{-1}), V_{cm} and V_{om} are the carboxylation and oxygenation rates of mesophyll Rubisco (mol CO_2 or O_2 m^{-2} s^{-1}), and n_L and n_C are the ATP coupling efficiencies of LEF and CEF1, respectively (mol ATP mol^{-1} e^-). For the Type I C_3-C_4 bundle sheath, the analogous expressions are given by:

$$J_{P680s} = 4 \cdot V_{cs} + 4 \cdot V_{os} \tag{1c}$$

$$J_{P700s} = (3 \cdot V_{cs} + 3.5 \cdot V_{os} - J_{P680s} \cdot n_L)/n_C + J_{P680s} \tag{1d}$$

where J_{P680s} is the total rate of LEF through bundle sheath PS II (mol e^- m^{-2} s^{-1}), J_{P700s} is the total rate of LEF and CEF1 through bundle sheath PS I (mol e^- m^{-2} s^{-1}), and V_{cs} and V_{os} are the carboxylation and oxygenation rates of bundle sheath Rubisco (mol CO_2 or O_2 m^{-2} s^{-1}). For the C_4 mesophyll, the corresponding expressions are:

$$J_{P680m} = 2 \cdot V_{pm} \tag{2a}$$

$$J_{P700m} = 2 \cdot V_{pm} \cdot (1 - n_L/n_C + 1/n_C) \tag{2b}$$

where V_{pm} is the mesophyll activity of PEPC (mol CO_2 m^{-2} s^{-1}). In Eqns. 2a-2b, the partitioning between LEF and CEF1 in the mesophyll reflects the requirement for 2 ATP and 1 NADPH to drive the NADP-ME type C_4 cycle. For the C_4 bundle sheath, the corresponding expressions are:

$$J_{P680s} = 4 \cdot V_{cs} + 4 \cdot V_{os} - 2 \cdot V_{ds} \tag{2c}$$

$$J_{P700s} = (3 \cdot V_{cs} + 3.5 \cdot V_{os} - J_{P680s} \cdot n_L)/n_C + J_{P680s} \tag{2d}$$

where V_{ds} is the bundle sheath activity of NADP-ME (mol CO_2 m^{-2} s^{-1}). In Eqns. 2c-2d, the partitioning between LEF and CEF1 in the bundle sheath reflects the energetic requirements to drive the PCR and PCO cycles as well as the partial suppression of LEF due to NADPH production by NADP-ME.

Steady-state gas-exchange

We continue by defining expressions that link the activity of carbon metabolism to gas-exchange. For a C_3, Type I C_3-C_4, or C_4 leaf, a general expression for the net assimilation rate (A) is given by the sum of net CO_2 assimilation in the mesophyll (A_m) and the bundle sheath (A_s):

$$A = A_m + A_s \tag{3a}$$

$$A_m = V_{cm} - 0.5 \cdot V_{om} - R_m \tag{3b}$$

$$A_s = V_g + V_p - L \tag{3c}$$

where A, A_m, and A_s can have units of mol assimilated C m^{-2} s^{-1} or mol CO_2 m^{-2} s^{-1}. In Eqn. 3c, V_g is the net rate at which the glycine shuttle transfers assimilated C from the mesophyll into the bundle sheath (mol C m^{-2} s^{-1}), V_p is the net rate at which the malate shuttle transfers assimilated C from the mesophyll into the bundle sheath (mol C m^{-2} s^{-1}), and L is the rate at which CO_2 leaks out of the bundle sheath via diffusion (mol CO_2 m^{-2} s^{-1}). The rate of the glycine shuttle is determined by mesophyll Rubisco oxygenase activity that is coupled to bundle sheath GDC activity, and the rate of the malate shuttle is based on mesophyll PEPC activity that is coupled to bundle sheath NADP-ME activity:

$$V_g = 0.5 \cdot V_{om} - V_{gm} \tag{4a}$$

$$V_p = V_{pm} - V_{dm} \tag{4b}$$

where V_{gm} is the rate of GDC activity in the mesophyll (mol CO_2 m^{-2} s^{-1}), V_{pm} is the rate of carboxylation via mesophyll PEPC (mol CO_2 m^{-2} s^{-1}) and V_{dm} is mesophyll C_4-decarboxylase activity that results in futile cycling (mol CO_2 m^{-2} s^{-1}). Combining Eqns. 3-4 then yields a second expres-

sion for the overall net CO_2 assimilation rate (A):

$$A = V_{cm} - V_{gm} + V_{pm} - V_{dm} - L - R_m \tag{5}$$

which can represent photosynthesis in C_3, any of the various C_3-C_4, or C_4 plants. If all of the Rubisco is localized to the mesophyll, this expression reduces to the regular C_3 expression described by Farquhar et al. (1980). If some of the Rubisco and all of the GDC are localized to the bundle sheath, the reduced expression is equivalent to the Type I C_3-C_4 expression described by von Caemmerer (1989). If all of the Rubisco is localized to the bundle sheath and there is no futile C_4 cycling, the reduced expression is equivalent to the C_4 expression described by Berry and Farquhar (1978). In the dark, the leak rate L is equivalent to the rate of bundle sheath respiration, R_s.

Linking electron transport with gas-exchange

The electron transport rates can now be linked directly to the net CO_2 exchange rates using the relationship:

$$V_o/V_c = \frac{1}{S} \cdot \frac{O}{C} = \frac{K_c \cdot k_o \cdot O}{k_c \cdot K_o \cdot C} \tag{6}$$

where O and C are the pO_2 and pCO_2 in the chloroplast (bar), S is the specificity of Rubisco for carboxylation relative to oxygenation (mol CO_2 mol^{-1} O_2), k_c and k_o are the catalytic constants of Rubisco for CO_2 and O_2 (mol CO_2 or O_2 mol^{-1} sites s^{-1}, and K_c and K_o are the Michaelis-Menten constants for CO_2 and O_2 (bar). For the C_3 and Type I C_3-C_4 mesophyll, combining Eqns. 1a, 1b, 5, and 6 gives:

$$J_{P680m} = \frac{J_{P700m}}{1 - \frac{n_L}{n_C} + \left[\frac{3 + 7 \cdot O_m/(2 \cdot S \cdot C_m)}{(4 + 4 \cdot O_m/(S \cdot C_m)) \cdot n_C}\right]} \tag{7a}$$

$$A_m = \frac{J_{P680m} \cdot [1 - O_m/(2 \cdot S \cdot C_m)]}{4 \cdot [1 + O_m/(S \cdot C_m)]} - R_m \tag{7b}$$

where O_m and C_m are the mesophyll pO_2 and pCO_2 (bar). For the Type I C_3-C_4 bundle sheath, the analogous expressions are derived by combining Eqns. 1c, 1d, 5, and 6:

$$J_{P680s} = \frac{J_{P700s}}{1 - \frac{n_L}{n_C} + \left[\frac{3 + 7 \cdot O_s/(2 \cdot S \cdot C_s)}{(4 + 4 \cdot O_s/(S \cdot C_s)) \cdot n_C}\right]} \tag{8a}$$

$$A_s = \frac{J_{P680s} \cdot [1 - O_s/(2 \cdot S \cdot C_s)]}{4 \cdot [1 + O_s/(S \cdot C_s)]} - R_s \tag{8b}$$

where O_s and C_s are the bundle sheath pO_2 and pCO_2 (bar). For the C_4 mesophyll, the expressions for linear electron flow through PS II and the net CO_2 assimilation reduce to:

$$J_{P680m} = \frac{J_{P700m}}{1 - \frac{n_L}{n_C} + \frac{1}{n_C}} \tag{9a}$$

$$A_m = -R_m \tag{9b}$$

which are derived from Eqns. 2a, 2b, and 5. For the C_4 bundle sheath, the corresponding expressions for the linear electron flow through PS II and the net CO_2 assimilation expand to:

$$J_{P680s} = \frac{J_{P700s} - J_{P680m} \cdot \left[\frac{3 + 7 \cdot O_s/(2 \cdot S \cdot C_s)}{(4 + 4 \cdot O_s/(S \cdot C_s)) \cdot n_C}\right]}{1 - \frac{n_L}{n_C} + \left[\frac{3 + 7 \cdot O_s/(2 \cdot S \cdot C_s)}{(4 + 4 \cdot O_s/(S \cdot C_s)) \cdot n_C}\right]} \tag{10a}$$

$$A_s = \frac{[J_{P680s} + J_{P680m}] \cdot [1 - O_s/(2 \cdot S \cdot C_s)]}{4 \cdot [1 + O_s/(S \cdot C_s)]} - R_s \tag{10b}$$

which are derived from Eqns. 2c, 2d, 5, and 6. Under different environmental conditions, Eqns. 7-10 can be solved using the expressions in Appendices II, III, IV, and V.

Appendix II: Pure light-limited state

Expressions for net CO_2 assimilation (A_{mj} and A_{sj})

In a pure light-limited state, the net CO_2 assimilation rates of the mesophyll and bundle sheath (A_{mj}, A_{sj}) are kinetically controlled by the maximum activity of Cyt b_6f and the excitation balance of PS I and PS II. For the C_3, Type I C_3-C_4, and C_4 cases, the light-limited rates of PS I electron transport in the mesophyll and bundle sheath (J_{P700mj}, J_{P700sj}) are given by:

$$J_{P700mj} = \frac{V_{mmax\ (CB6F)} \cdot Q}{\left[\frac{V_{mmax\ (CB6F)}}{\alpha_{1m} \cdot K_{P1}/(K_{P1} + K_{D1} + K_{F1})}\right] + Q} \tag{11a}$$

$$J_{P700sj} = \frac{V_{smax\ (CB6F)} \cdot Q}{\left[\frac{V_{smax\ (CB6F)}}{\alpha_{1s} \cdot K_{P1}/(K_{P1} + K_{D1} + K_{F1})}\right] + Q} \tag{11b}$$

where Q is the photosynthetically active radiation incident on the leaf (mol incident PPFD m^{-2} s^{-1}), α_{1m} and α_{1s} are the absorbance cross-sections associated with the PS I bed in the mesophyll and bundle sheath (mol PPFD absorbed by PS I mol^{-1} incident PPFD), $K_{P1}/(K_{P1} + K_{D1} + K_{F1})$ is the maximum photochemical yield of PS I (mol energy dissipated

https://doi.org/10.1007/s00442-021-05062-y

mol^{-1} energy absorbed), and $V_{mmax\ (CB6F)}$ and $V_{smax\ (CB6F)}$ are the maximum activities of Cyt b_6f in the mesophyll and bundle sheath (mol e^- m^{-2} s^{-1}).

Since the total electron flow through PS I may be comprised of any combination of linear and cyclic components, it represents the upper limit for linear electron flow through PS II (J_{P680mj}, J_{P680sj}). For the C_3, Type I C_3-C_4, and C_4 mesophyll, Eqn. 11a can be substituted into Eqn. 7a-7b and Eqn. 9a-9b to calculate the rate of electron flow through PS II and the corresponding rate of net CO_2 assimilation in the mesophyll (A_{mj}) from Q, O_m, and C_m. For the Type I C_3-C_4 and C_4 bundle sheath, Eqn. 11b can be substituted into Eqn. 8a-8b and Eqn. 10a-10b to calculate the rate of electron flow through PS II, and the corresponding rate of net CO_2 assimilation in the bundle sheath (A_{sj}) from Q, O_s, and C_s. The expressions for C_s and O_s are given below.

Expressions for bundle sheath CO_2 (C_s)

For the Type I C_3-C_4 and C_4 cases, the steady-state CO_2 concentration of the bundle sheath is a balance between CO_2 supply (a function of glycine or malate shuttling) and demand (a function of net assimilation and diffusive leakage). However, the supply of CO_2 to the bundle sheath depends on the state of the mesophyll. For the Type I C_3-C_4 case, the light-limited rate of glycine shuttling is given by:

$$V_{gj} = \frac{J_{P680mj} \cdot [O_m/(2 \cdot S \cdot C_m)]}{4 \cdot [1 + O_m/(S \cdot C_m)]} \tag{12a}$$

which is dependent on the energy supply in the mesophyll. For the C_4 case, the light-limited rate of malate shuttling is given by:

$$V_{pj} = \frac{J_{P700mj}}{2 \cdot \left[1 - \frac{n_L}{n_C} + \frac{1}{n_C}\right]} \tag{12b}$$

which is also dependent on the energy supply in the mesophyll. On the demand side, the rate of diffusive leakage (L) is given by:

$$L = \frac{g_{bs} \cdot (C_s - C_m)}{P_{tot}} \tag{13}$$

where g_{bs} is the conductance of the bundle sheath cell wall to CO_2 (mol CO_2 m^{-2} s^{-1}), C_s - C_m is the CO_2 partial pressure gradient between the bundle sheath and the mesophyll (bar), and P_{tot} is the total atmospheric pressure (bar). Writing a conservation equation and rearranging yields:

$$C_s = C_m + [(V_{gj} + V_{pj} - A_{sj}) \cdot P_{tot}/g_{bs}] \tag{14}$$

where C_s is represented as a function of A_{sj}.

Expressions for bundle sheath O_2 (O_s)

Just as for CO_2, the steady-state O_2 concentration of the bundle sheath is a balance between O_2 supply (a function of splitting of water at PS II) and demand (a function of Rubisco oxygenase activity, respiration, and diffusive leakage). For the Type I C_3-C_4 case, we specify that net O_2 evolution is proportional to net CO_2 assimilation (A_s). For the C_4 case, we specify that net O_2 evolution is proportional to net CO_2 assimilation less the O_2 evolution that is suppressed by NADP-ME activity ($A_s - V_p/2$). For the diffusive leak, L_o is given by:

$$L_o = \frac{g_o \cdot (O_s - O_m)}{P_{tot}} \tag{15a}$$

where g_o is the conductance of the bundle sheath cell wall to O_2 (mol O_2 m^{-2} s^{-1}), and O_s - O_m is the O_2 partial pressure gradient between the bundle sheath and the mesophyll (bar). The conductance of the bundle sheath cell wall to O_2 is linked to the conductance of the bundle sheath cell wall to CO_2 by:

$$g_o = g_{bs} \cdot \frac{D_o \cdot H_o}{D_c \cdot H_c} \tag{15b}$$

where D_o and D_c are the diffusivities of O_2 and CO_2 in water (m^2 s^{-1}), and H_o and H_c are the solubilities of O_2 and CO_2 in water (bar^{-1}). Writing a conservation equation and rearranging yields the steady-state O_2 concentration of the bundle sheath as:

$$O_s = O_m + \frac{(A_{sj} - V_{pj}/2) \cdot P_{tot}}{g_o} \tag{16}$$

where O_s is represented as a function of A_{sj}.

Appendix III: Pure light-saturated state

Expressions for net CO_2 assimilation (A_{mc} and A_{sc})

In a pure light-saturated state, the CO_2 assimilation rates of the mesophyll and bundle sheath (A_{mc}, A_{sc}) are kinetically controlled by the maximum activity of Rubisco and/or

PEPC. For A_{mc} and A_{sc}, the rate equations are given by:

$$A_{mc} = \frac{V_{mmax} \cdot [C_m - O_m/(2 \cdot S)]}{K_c \cdot (1 + O_m/K_o) + C_m} - R_m \quad (17a)$$

$$A_{sc} = \frac{V_{smax} \cdot [C_s - O_s/(2 \cdot S)]}{K_c \cdot (1 + O_s/K_o) + C_s} - R_s \quad (17b)$$

where V_{mmax} and V_{smax} are the maximum carboxylase activities of Rubisco for the mesophyll and bundle sheath (mol CO_2 m^{-2} s^{-1}). For the C_3 and Type I C_3-C_4 mesophyll, Eqn. 17a can be solved for A_{mc} directly from O_m and C_m, and then substituted into Eqn. 7a-7b to calculate the corresponding rates of electron flow through mesophyll PS II and PS I (J_{P680mc}, J_{P700mc}). For the Type I C_3-C_4 and C_4 bundle sheath, Eqn. 17b can be solved for A_{sc} from O_s and C_s, and then substituted into Eqn. 8a-8b and Eqn. 10a-10b to calculate the corresponding rates of electron flow through bundle sheath PS II and PS I (J_{P680sc}, J_{P700sc}). Expressions for calculation of C_s and O_s are given below.

Expressions for bundle sheath CO_2 and O_2 (C_s and O_s)

As before, the CO_2 supply depends on the state of the mesophyll. For the Type I C_3-C_4 case, the light-saturated rate of glycine shuttling (V_{gc}) is given by:

$$V_{gc} = \frac{V_{mmax} \cdot [O_m/(2 \cdot S)]}{K_c \cdot (1 + O_m/K_o) + C_m} \quad (18a)$$

where all of the terms have been defined previously. For the C_4 case, the light-saturated rate of malate shuttling (V_{pc}) is given by:

$$V_{pc} = \frac{V_{pmax} \cdot C_m}{K_p + C_m} \quad (18b)$$

where V_{pmax} is the maximum mesophyll activity of PEPC (mol CO_2 m^{-2} s^{-1}), and K_p is the Michaelis-Menten constant of PEPC for CO_2 (bar).

The expression for the partial pressure of CO_2 in the bundle sheath is then given by:

$$C_s = C_m + \frac{(V_{gc} + V_{pc} - A_{sc}) \cdot P_{tot}}{g_{bs}} \quad (19)$$

where C_s is represented as a function of A_{sc}. The corresponding expression for the partial pressure of O_2 in the bundle sheath is given by:

$$O_s = O_m + \frac{(A_{sc} - V_{pc}/2) \cdot P_{tot}}{g_o} \quad (20)$$

where O_s is also represented as a function of A_{sc}.

Appendix IV: Mixed light-limited/light-saturated states

For the Type I C_3-C_4 and C_4 cases, we also define two 'mixed states': one in which the mesophyll is light-limited while the bundle sheath is light-saturated, and the other in which the mesophyll is light-saturated while the bundle sheath is light-limited. In the first mixed state, the CO_2 supply to the bundle sheath is controlled by the light-limited rates of glycine shuttling (V_{gj}) or malate shuttling (V_{pj}) while the rate of net CO_2 assimilation in the bundle sheath is controlled by the light-saturated rate of Rubisco activity (A_{sc}). In this state, the partial pressures of CO_2 and O_2 in the bundle sheath are given by:

$$C_s = C_m + \frac{(V_{gj} + V_{pj} - A_{sc}) \cdot P_{tot}}{g_{bs}} \quad (21a)$$

$$O_s = O_m + \frac{(A_{sc} - V_{pj}/2) \cdot P_{tot}}{g_o} \quad (21b)$$

which are solved with substitution from Eqns. 12a, 12b, and 17b. In the second mixed state, the CO_2 supply to the bundle sheath is controlled by the light-saturated rates of glycine shuttling (V_{gc}) or malate shuttling (V_{pc}) while the rate of net CO_2 assimilation in the bundle sheath is controlled by the light-limited rate of Rubisco activity (A_{sj}). In this state, the partial pressures of CO_2 and O_2 in the bundle sheath are given by:

$$C_s = C_m + \frac{(V_{gc} + V_{pc} - A_{sj}) \cdot P_{tot}}{g_{bs}} \quad (22a)$$

$$O_s = O_m + \frac{(A_{sj} - V_{pc}/2) \cdot P_{tot}}{g_o} \quad (22b)$$

which are solved with substitution from Eqns. 8a, 8b, 11b, and 18a for the Type I C_3-C_4 case and with substitution from Eqns. 10a, 10b, 11b, and 18b for the C_4 case.

Appendix V: Solution for pure and mixed states

Once the potential limiting states are defined, the model is solved in three steps. In the first step, the realized net CO_2

https://doi.org/10.1007/s00442-021-05062-y

assimilation rate of the mesophyll is calculated as:

$$A_m = min\{A_{mj}, A_{mc}\} \quad (23)$$

where $min\{\}$ denotes the minimum of the potential light-limited (A_{mj}) and light-saturated (A_{mc}) rates. In the second step, the realized net CO_2 assimilation rate of the bundle sheath is calculated. We first calculate the potential light-limited net CO_2 assimilation rate using:

$$A_{sj} = \begin{cases} f(V_{gj}, V_{pj}), & \text{if} \quad A_m = A_{mj} \\ f(V_{gc}, V_{pc}), & \text{if} \quad A_m = A_{mc} \end{cases} \quad (24a)$$

to account for the pure state where the mesophyll and bundle sheath are both light-limited as well as the mixed state where the mesophyll is light-saturated and the bundle sheath is light-limited. We then calculate the potential light-saturated net CO_2 assimilation rate using:

$$A_{sc} = \begin{cases} f(V_{gj}, V_{pj}), & \text{if} \quad A_m = A_{mj} \\ f(V_{gc}, V_{pc}), & \text{if} \quad A_m = A_{mc} \end{cases} \quad (24b)$$

to account for the mixed state where the mesophyll is light-limited and the bundle sheath is light-saturated as well as the pure state where the mesophyll and bundle sheath are both light-saturated. The realized net CO_2 assimilation rate of the bundle sheath is then given by:

$$A_s = min\{A_{sj}, A_{sc}\} \quad (24c)$$

where $min\{\}$ denotes the minimum of the potential light-limited (A_{sj}) and light-saturated (A_{sc}) rates. In the third step, the mesophyll and bundle sheath rates from Eqns. 23 and 24c are combined:

$$A = A_m + A_s \quad (25)$$

which gives the overall rate of net CO_2 assimilation.

Once the limiting states have been identified, the expressions in Johnson and Berry (2021) can be used to derive many other aspects of the internal state of the electron transport system (e.g., the photochemical yields of PS II and PS I, the redox poise of the mobile electron carrier pools in the intersystem chain, the level of non-photochemical quenching of PS II, and the state of photosynthetic control of Cyt b_6f). These internal states then provide a basis for simulating observable fluorescence- and absorbance-based signals. Example calculations are provided in the model code. Note that for simplicity and clarity, all of these calculations assume that: (i) LEF is always accompanied by the amount of CEF1 required to balance the energy supply with the demands of carbon metabolism; and (ii) the absorption cross-sections of PS II and PS I are static rather than dynamic. See Johnson and Berry (2021) for discussion of alternatives.

References

Berry JA, Farquhar GD (1978) The CO_2 concentrating function of C_4 photosynthesis: A biochemical model. In: Hall D, Coombs J, Goodwin T (eds) Proceedings of the Fourth International Congress on Photosynthesis, Reading, England. The Biochemical Society, London, pp 119–131

Farquhar GD, von Caemmerer S, Berry JA (1980a) A biochemical model of photosynthetic CO_2 assimilation in leaves of C_3 species. Planta 149:78–90. https://doi.org/10.1007/bf00386231

Johnson JE, Berry JA (2021) The role of Cytochrome b_6f in the control of steady-state photosynthesis: a conceptual and quantitative model. Photosynth Res 148:101–136. https://doi.org/10.1007/s11120-021-00840-4

von Caemmerer S (1989) A model of photosynthetic CO_2 assimilation and carbon-isotope discrimination in leaves of certain C_3-C_4 intermediates. Planta 178:463–474. https://doi.org/10.1007/bf00963816
